# Vasopressin and Breathing: Review of Evidence for Respiratory Effects of the Antidiuretic Hormone

**DOI:** 10.3389/fphys.2021.744177

**Published:** 2021-10-26

**Authors:** Michał Proczka, Jacek Przybylski, Agnieszka Cudnoch-Jędrzejewska, Ewa Szczepańska-Sadowska, Tymoteusz Żera

**Affiliations:** ^1^Department of Experimental and Clinical Physiology, Doctoral School, Medical University of Warsaw, Warsaw, Poland; ^2^Department of Biophysics, Physiology, and Pathophysiology, Laboratory of Centre for Preclinical Research, Medical University of Warsaw, Warsaw, Poland; ^3^Department of Experimental and Clinical Physiology, Laboratory of Centre for Preclinical Research, Medical University of Warsaw, Warsaw, Poland

**Keywords:** antidiuretic hormone, respiration, cardiovascular system, carotid body, sympathetic nervous system, circumventricular organs, brainstem, paraventricular nucleus of the hypothalamus

## Abstract

Vasopressin (AVP) is a key neurohormone involved in the regulation of body functions. Due to its urine-concentrating effect in the kidneys, it is often referred to as antidiuretic hormone. Besides its antidiuretic renal effects, AVP is a potent neurohormone involved in the regulation of arterial blood pressure, sympathetic activity, baroreflex sensitivity, glucose homeostasis, release of glucocorticoids and catecholamines, stress response, anxiety, memory, and behavior. Vasopressin is synthesized in the paraventricular (PVN) and supraoptic nuclei (SON) of the hypothalamus and released into the circulation from the posterior lobe of the pituitary gland together with a C-terminal fragment of pro-vasopressin, known as copeptin. Additionally, vasopressinergic neurons project from the hypothalamus to the brainstem nuclei. Increased release of AVP into the circulation and elevated levels of its surrogate marker copeptin are found in pulmonary diseases, arterial hypertension, heart failure, obstructive sleep apnoea, severe infections, COVID-19 due to SARS-CoV-2 infection, and brain injuries. All these conditions are usually accompanied by respiratory disturbances. The main stimuli that trigger AVP release include hyperosmolality, hypovolemia, hypotension, hypoxia, hypoglycemia, strenuous exercise, and angiotensin II (Ang II) and the same stimuli are known to affect pulmonary ventilation. In this light, we hypothesize that increased AVP release and changes in ventilation are not coincidental, but that the neurohormone contributes to the regulation of the respiratory system by fine-tuning of breathing in order to restore homeostasis. We discuss evidence in support of this presumption. Specifically, vasopressinergic neurons innervate the brainstem nuclei involved in the control of respiration. Moreover, vasopressin V1a receptors (V1aRs) are expressed on neurons in the respiratory centers of the brainstem, in the circumventricular organs (CVOs) that lack a blood-brain barrier, and on the chemosensitive type I cells in the carotid bodies. Finally, peripheral and central administrations of AVP or antagonists of V1aRs increase/decrease phrenic nerve activity and pulmonary ventilation in a site-specific manner. Altogether, the findings discussed in this review strongly argue for the hypothesis that vasopressin affects ventilation both as a blood-borne neurohormone and as a neurotransmitter within the central nervous system.

## Introduction

Vasopressin (AVP), also known as antidiuretic hormone, is a neurohormone critically involved in maintaining body homeostasis. It is synthesized in discrete nuclei of the hypothalamus and transported to the posterior lobe of the pituitary gland, from where it is released into the circulation in response to increase of extracellular fluid osmolality. In the bloodstream, AVP is paramount for maintaining water balance thanks to its renal action resulting in water reabsorption and urine concentration. The other stimuli for AVP release include hypovolemia, hypotension, hypoxia, hypoglycemia, strenuous exercise, and angiotensin II (Ang II; Szczepanska-Sadowska et al., 2017). The same stimuli also promote increases in pulmonary ventilation and are often associated with life-threatening conditions (Doerschug et al., 2010; Frier, 2014; Thompson et al., 2016; Convertino et al., 2019; Lüning et al., 2019).

In addition to its effects in the kidney, AVP exerts numerous extra-renal effects, including circulatory, nervous, endocrine, metabolic, and behavioral ones, which are discussed in detail in recent reviews (Szczepanska-Sadowska et al., 2017; Japundžić-Žigon et al., 2020). Together, these AVP-mediated responses counteract disturbances of the body homeostasis and help in adjusting body function to internal and environmental stressors. Along with the control of circulatory and nervous system, the precise regulation of the respiratory system is also critical for the homeostatic adjustments. It is important to realize that changes in pulmonary ventilation fulfil their adaptive role only when they are matched with parallel changes in cardiac output and body metabolism. In this review, we summarize the most important studies analyzing regulation of AVP release and discuss the effects of this neuropeptide on the respiration under physiological and pathophysiological conditions, acting both as a blood-borne neurohormone and as a neurotransmitter within the central nervous system.

## Physiology of Vasopressin

### Vasopressin: Synthesis, Receptors, and Release

Vasopressin is synthesized in the form of pre-pro-AVP, which is processed into AVP, neurophysin II and C-terminal fragment of pre-pro-AVP known as copeptin. The neurohormone is produced predominantly in the paraventricular (PVN) and supraoptic nuclei (SON) of the hypothalamus in two histologically and functionally distinct pools of neurons – magnocellular cells projecting to the posterior pituitary and parvocellular cells projecting to the median eminence and extrahypothalamic brain structures, especially to the limbic system (Buijs, 1978; Dumais and Veenema, 2016) and the brainstem (Buijs, 1978; Kc and Dick, 2010; Kc et al., 2010). Vasopressin is released into the circulation together with copeptin from the axonal terminals of the magnocellular neurons located in the posterior lobe of the pituitary gland (neurohypophysis; Schrier et al., 1979; Szczepanska-Sadowska et al., 2017; Bichet, 2019). Vasopressin is also released from the nerve terminals of the PVN parvocellular cells in the median eminence into the hypothalamic-pituitary circulation, through which AVP reaches anterior lobe of the pituitary gland and promotes ACTH release (Gonzalez-Luque et al., 1970; Lee et al., 2015). Besides hypothalamic synthesis in the PVN and the SON, AVP, or AVP mRNA is locally expressed in peripheral organs, such as the adrenal medulla and the heart (Nussey et al., 1987; Hupf et al., 1999; Takeda et al., 2002). Measurements of plasma concentrations of AVP are highly variable due to binding of the neurohormone to platelets and its short biological half-life (Nickel et al., 2012; Bankir et al., 2017). Copeptin, co-released with AVP in equimolar quantities, is very stable and may serve as a biomarker of AVP release (Morgenthaler et al., 2006; Bankir et al., 2017).

Vasopressin acts *via* three subtypes of receptors, which belong to the G-protein coupled receptors: V1aR, V1bR, and V2R. Vasopressin released into the circulation exerts its cardiovascular effects mainly through V1aRs, which mediate vasoconstriction and increase in vascular resistance in most of the vascular beds (Szczepanska-Sadowska et al., 2017; Japundžić-Žigon et al., 2020), and complex effects in the coronary circulation and cardiac hemodynamics (Pelletier et al., 2014). Binding of AVP to V1aRs expressed on thrombocytes stimulates procoagulant activity of platelets (Launay et al., 1987; Horstman et al., 1995; Colucci et al., 2014).

Vasopressin also exerts numerous endocrine effects mediated by V1aRs and V1bRs that include regulation of insulin and glucagon release from the pancreatic islets, release of catecholamines in the adrenal medulla and glucocorticoids in the adrenal cortex, and stimulation of corticotropin release from the pituitary gland (Nussey et al., 1987; Aguilera and Rabadan-Diehl, 2000; Takeda et al., 2002; Szczepanska-Sadowska et al., 2017; Mohan et al., 2019). In addition, AVP released into the circulation promotes gluconeogenesis and glycogenolysis in the liver and lipid metabolism in the fat tissue (Nakamura et al., 2017).

Vasopressin plays a critical role in the regulation of water-electrolyte balance *via* its V2Rs in the kidney, which depends on upregulation of the aquaporin 2 with resultant water trafficking in the apical membrane of the principal cells of the collecting duct (Wade et al., 1981; Schrier, 2008; Bankir et al., 2017).

Besides the systemic effects of AVP in the bloodstream, the neurohormone serves as a peptidergic neurotransmitter with both synaptic and “volume” mode of neurotransmission (Landgraf and Neumann, 2004). Vasopressin is released in the central nervous system from the nerve terminals of vasopressinergic neurons, whose cell bodies are located in the parvocellular division of the PVN. Up to 40% of the parvocellular cells of the PVN synthesize AVP and project to the brainstem and the spinal cord, where they terminate on sympathetic neurons (Pyner, 2009; Nunn et al., 2011). Vasopressinergic neurons exert either V1aR-dependent sympthoexcitatory effects accompanied by a rise in arterial blood pressure, or sympathoinhibition with activation of the parasympathetic system and sensitization of the arterial baroreflex (Szczepanska-Sadowska et al., 2017; Japundžić-Žigon et al., 2020).

In addition to the central cardiovascular effects, both V1aRs and V1bRs participate in the regulation of mood, anxiety, aggression, pain, cognitive processes and memory, and adaptation to stress (Aguilera and Rabadan-Diehl, 2000; Szczepanska-Sadowska et al., 2017). Emerging evidence indicates that V1bRs may serve as autoreceptors on the vasopressinergic neurons in the hypothalamus (Corbani et al., 2018). Recently, it has been shown that V1bRs may be involved in SARS-CoV-2 infection by participating in the endocytosis of the virus’ particles (Yeung et al., 2021). The tissue distribution of AVP receptors, their diverse biological functions, as well as agonists and antagonists are discussed in detail elsewhere (Koshimizu et al., 2012; Szczepanska-Sadowska et al., 2017). The respiratory actions of AVP are discussed in detail in the following sections.

### Vasopressin Release and Respiration: Coincidence or Relationship?

**Figure 1:**
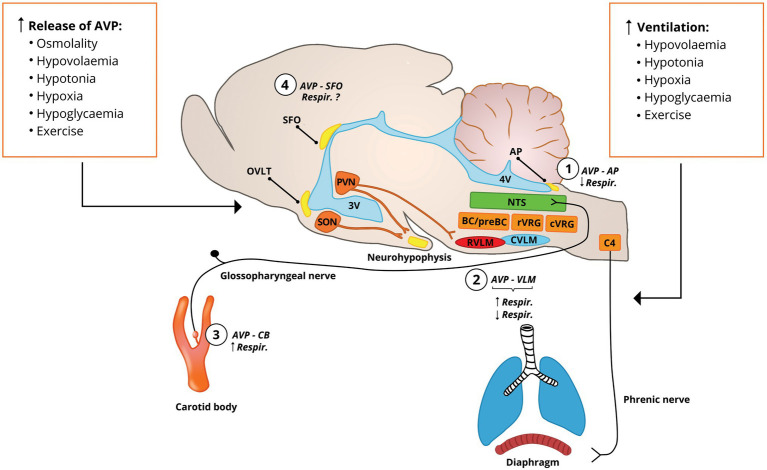
Stimuli that cause AVP release into the bloodstream and activation of the vasopresinergic PVN neurons also increase the pulmonary ventilation. AVP in the bloodstream accesses the circumventricular organs (SFO, AP, and OVLT) that lack the blood-brain barrier and the carotid body at the carotid bifurcation. Vasopressinergic PVN neurons project to cardiovascular (RVLM) and respiratory (preBC/BC, rVRG, and C4 phrenic nucleus) centers, suggesting their involvement in the respiratory control (Kc et al., 2002a, 2010). (1) AVP at the AP inhibits phrenic nerve activity (Yang et al., 2006); (2) vasopressinergic projections to the rVRG tonically stimulate respiratory activity (Kc et al., 2010) and AVP applied into the rVRG or pre-Bötzinger complex stimulates activity of diaphragm (Kc et al., 2002a, 2010), which is accompanied by increase in arterial blood pressure; this is in contrast to findings indicating that AVP administered into the rVRG inhibits phrenic nerve activity with and without changes in arterial blood pressure (Chuang et al., 2003, 2005; Cheng et al., 2004); (3) AVP locally administered into the carotid bifurcation slightly increases ventilation (Żera et al., 2018); and (4) vasopressin receptors are expressed in the SFO (Ostrowski et al., 1994), and electric stimulation of SFO increases respiratory activity (Ferguson et al., 1989), but the respiratory effects of AVP acting at SFO have not been determined. AP, area postrema; AVP, vasopressin; BC, Bötzinger complex; C4, phrenic nuclei; CVLM, caudal ventrolateral medulla; cVRG, caudal ventral respiratory group; NTS, nucleus of the solitary tract; OVLT, organum vasculosum of the lamina terminalis; preBC, pre-Bötzinger complex; PVN, paraventricular nucleus of the hypothalamus; RVLM, rostral ventrolateral medulla; rVRG, rostral ventral respiratory group; SFO, subfornical organ; SON, supraoptic nucleus; and VLM, ventral lateral medulla.

The evidence discussed below indicates that stimuli that activate vasopressinergic neurons and increase AVP levels also affect respiratory activity (Figure 1). This raises a question whether release of AVP and changes in respiration in response to these stimuli are coincidental, or if there is a functional relationship between AVP and the regulation of the respiratory system. Next, we discuss studies showing increased release of AVP and its surrogate marker copeptin under medical conditions accompanied by respiratory disturbances. Then, we analyze the evidence for AVP involvement in the regulation of respiratory system.

#### Regulation of Vasopressin Release

Activation of the vasopressinergic neurons and release of AVP into the circulation occurs in response to osmotic and non-osmotic stimuli. The magno- and parvocellular vasopressinergic cells of the hypothalamus are distinctly activated by various stimuli, with magnocellular cells being mostly activated by increase in sodium ion concentration and osmolality, hypovolemia, hypotonia, and hypoxia, while the parvocellular cells are more sensitive to various stressors, such as pain, injury, and psychological stress (Aguilera et al., 2008; Ueta et al., 2011; Bankir et al., 2017; Szczepanska-Sadowska et al., 2017). Next, we discuss main stimuli for AVP release and how they affect the respiratory system.

#### Osmotic - Dependent AVP Release

Vasopressin is released into the circulation in response to increase in plasma osmolality and sodium ion concentration in directly proportional manner (Verney, 1947; Quillen and Cowley, 1983; Thornton et al., 1986; Baylis, 1987; Verbalis, 2007). In young healthy men, plasma AVP concentration starts to increase at plasma osmolality of 285mOsm/kg, and continues to rise till values of osmolality exceed 310mOsm/kg (Baylis and Robertson, 1980; Baylis, 1987). The changes in osmolality are detected in the organum vasculosum of the lamina terminalis (OVLT), in the subfornical organ (SFO), and in the magnocellular neurons of the PVN and the SON (Anderson et al., 2000, 2001; Verbalis, 2007; Bankir et al., 2017; Szczepanska-Sadowska et al., 2017). The changes of extracellular fluid osmolality are mostly affected by changes in sodium ion concentration; however, other osmotically active substances (e.g., mannitol) that affect cell volume also trigger AVP release (Verbalis, 2007). On the other hand, osmotically active solutes freely passing through the cell membrane (e.g., urea), have much weaker effect on cell volume and AVP release (Verbalis, 2007). In addition, changes in activity of AVP neurons and subsequent release of AVP in the posterior pituitary lobe are regulated by availability of water and sensation of thirst and may precede actual changes in the extracellular fluid osmolality (Bankir et al., 2017).

Several studies indicate that plasma osmolality and concentration of sodium ions may contribute to the control of respiration. Specifically, hyperosmolality was shown to inhibit respiration and to reduce increase in pulmonary ventilation evoked by thermoregulatory panting or adjustments to acid-base balance disturbances in animals (Baker and Dawson, 1985; Kasserra et al., 1991; Kasserra and Jones, 1993) and humans (Senay, 1969; Moen et al., 2014). However, it should be noted that experiments in the *in situ* preparations revealed direct excitatory effects of hyperosmolality on the carotid sinus nerve and the phrenic nerve activity (Trzebski et al., 1978; Kasserra et al., 1991; O’Connor and Jennings, 2001; da Silva et al., 2019).

In contrast to hyperosmolality, acute hypoosmolality has been shown to stimulate breathing in conscious dogs (Anderson et al., 1990) and transiently in rats (O’Connor and Jennings, 2001). In women and men, hypoosmolality induced by ingestion of water also increases ventilation, although, this may be in part compensatory response to metabolic acidosis induced by ingestion of tap water (Moen et al., 2014).

Earlier experiments *in vivo* on cats have shown that perfusion of the carotid bodies with hypoosmotic solutions increases activity of the carotid sinus nerve (Gallego and Belmonte, 1979) that is associated with increase in breathing. This stimulatory effect of hypoosmolality, at least in part, may be attributed to activation of calcium currents and depolarization of chemoreceptors in the carotid body directly induced by hypoosmotic stimulus, as shown in a rat (Molnár et al., 2003). Another potential mechanism may depend on vasoconstriction of vessels supplying the carotid body, leading to decrease in the carotid body blood flow and activation of the chemosensitive glomus cells (Brognara et al., 2021). It has been shown that hypoosmolality causes constriction of the vascular smooth muscles in various vascular beds (Lang et al., 1995; Aoki et al., 2014), and this mechanism was postulated to be involved in the activation of carotid bodies and increased activity of the carotid sinus nerve (Gallego and Belmonte, 1979).

#### Non-osmotic - Dependent AVP Release

Vasopressinergic neurons can be activated and AVP secreted into the circulation in response to numerous non-osmotic stimuli. The most critical ones are hypovolemia and hypotension, physical exercise, hypoglycemia, hypoxia, and Ang II. Of note, these stimuli also affect the pulmonary ventilation and/or activate the carotid body and arterial chemoreflex.

##### Hypovolemia and Hypotension

Vasopressinergic neurons are tonically inhibited by sensory input from cardiopulmonary low-pressure mechanoreceptors and high-pressure arterial baroreceptors located in the cardiac atria and large systemic veins and the walls of the aortic arch and the carotid sinus, respectively (Schrier et al., 1979; Baylis, 1987; Norsk, 1989). Decrease in the central blood volume and arterial blood pressure leads to unloading of these two groups of cardiovascular mechanoreceptors, which in turn results in activation of the hypothalamic vasopressinergic neurons (Baylis, 1987; Szczepanska-Sadowska et al., 2017). Consequently, hypovolemia, hypotension, or hemorrhage lead to massive release of AVP and its surrogate marker copeptin into the circulation (Arnauld et al., 1977; Fyhrquist et al., 1981; Wehberg et al., 1991; Scott et al., 1994; Muller et al., 2007; Johnson et al., 2014; Bankir et al., 2017). It has been shown that in humans the arterial baroreceptors of the carotid sinus play a critical role in AVP release induced by decrease in arterial blood pressure (Norsk, 1989).

Hypovolemia and hypotension, caused by hemorrhage or pharmacologically induced vasodilation, lead to the increase in pulmonary ventilation (D’Silva et al., 1966; Ohtake and Jennings, 1992; Matsuoka et al., 1994; Walker and Jennings, 1998). Progressive reduction of the central blood volume raises ventilation in men (Convertino et al., 2009) and even small decreases in arterial pressure exert stimulatory effects on breathing in dogs (Ohtake and Jennings, 1992). Of note, under conditions of reduced cardiac output, this increase in pulmonary ventilation may serve as a pulmonary pump that by increasing the amplitude of changes in intrathoracic and intraabdominal pressures promotes venous return to the right cardiac ventricle and limits the decrease in stroke volume (Skytioti et al., 2018). The changes in respiratory activity are dependent on the intra-sinus pressure sensed by the arterial baroreceptors (Brunner et al., 1982). As the carotid baroreceptors are unloaded, the pulmonary ventilation increases, and vice versa, stimulation of the baroreceptors inhibits ventilation (Brunner et al., 1982). This inhibitory effect of the baroreflex on the respiratory system is particularly prominent during expiration in the *in situ* preparation in rat (Baekey et al., 2010) and is dependent on the respiratory drive in anesthetized rats (McMullan et al., 2009).

##### Physical Exercise

Physical activity is an acute stressor for the body homeostasis that robustly triggers release of AVP into the circulation proportionally to the intensity of exercise (Wade and Claybaugh, 1980; Popovic et al., 2019). Although exercise-induced hyperosmolality may be involved in the release of AVP (Takamata et al., 2000), during high-intensity physical activity the increase in plasma AVP (or its surrogate marker copeptin) may be present despite hyponatremia or lack of changes in plasma osmolality (Wade and Claybaugh, 1980; Inder et al., 1998; Hew-Butler et al., 2011), which indicates a non-osmotic release of AVP during intense physical exercise. Furthermore, it appears that prominent release of AVP in response to strenous exercise may lead to exercise-induced hyponatremia (Hew-Butler et al., 2017).

Physical activity is also the main physiological stimulus for the increase in pulmonary ventilation. In humans, both the high intensity dynamic exercise and the isometric muscle contractions may cause hyperventilation, especially at the end of exercise or during recovery phase, respectively (Imms and Mehta, 1989; Johnson et al., 1992). Thus, it is attractive to conjecture that AVP released during physical exercise may fine-tune respiratory responses and prevent excessive pulmonary ventilation, however, this requires further investigations.

##### Hypoglycemia

Insulin-induced hypoglycemia causes AVP release into the circulation both in animals (Plotsky et al., 1985; Berkenbosch et al., 1989; Caraty et al., 1990) and in human subjects (Baylis et al., 1981; Chiodera et al., 1992). The increased release of AVP in response to hypoglycemia appears to be independent of insulin (Chiodera et al., 1992).

The hypoglycemia-induced AVP release counteracts low plasma concentrations of glucose by gluconeogenic and glycogenolytic effects of AVP in the liver and on the AVP-induced activation of the hypothalamic-pituitary-adrenal cortex axis resulting in glucocorticoid release (Nakamura et al., 2017; Szczepanska-Sadowska et al., 2017). Both experimental studies in animals and clinical observations in humans suggest that carotid bodies may be involved in mediating the counter-regulatory response to decreased blood glucose (Koyama et al., 2000; Wehrwein et al., 2015; Kakall et al., 2019). Both in men and rats, hypoglycemia also induces pronounced hyperventilation, which depends on the release of humoral factors, such as adrenaline acting at the carotid bodies (Bin-Jaliah et al., 2004; Ward et al., 2007; Thompson et al., 2016).

##### Hypoxia

Hypoxia is a key trigger for the peripheral chemoreflex elicited from the carotid bodies, which results in increase of the pulmonary ventilation, sympathoexcitation, and arousal (Marshall, 1994; Kumar and Prabhakar, 2012; Zera et al., 2019).

Hypoxia is also an important chemical stimulus for AVP release (Iovino et al., 2013; Szczepanska-Sadowska et al., 2017). It increases neurohypophyseal blood flow and increases plasma concentration of AVP both in animals (Wang et al., 1984; Wilson et al., 1987; Hanley et al., 1988; Raff, 2011), and humans (Koller et al., 1991). The effect of hypoxia on AVP release depends on the sensory input from the carotid bodies (Levy, 1966; Wilson et al., 1987; Iovino et al., 2013). The rise of plasma AVP counteracts the hypoxia-induced vasodilation and helps in maintaining the peripheral vascular resistance (Walker, 1986; Louwerse and Marshall, 1993), the action that complements the hypoxia-evoked sympathoexcitation.

##### Angiotensin II

Angiotensin II is a potent hormone that causes vasoconstriction and activates the sympathetic nervous system, promotes renal reabsorption of sodium ions directly by acting on the renal tubules and indirectly through the aldosterone pathway (Sztechman et al., 2018). It is also the key hormonal factor causing AVP release (Fyhrquist et al., 1979; Flôr et al., 2018; Szczepanska-Sadowska et al., 2018). This allows for integration of water-electrolyte balance and regulation of osmolality and volume of extracellular fluid (Szczepańska-Sadowska, 1996).

Besides its cardiovascular, renal and sympathetic effects, Ang II also increases ventilation in dogs (Potter and McCloskey, 1979) and rats (Melo et al., 2020), however acute studies in humans show insignificant effect of Ang II on pulmonary ventilation (Bristow et al., 1971; Solaiman et al., 2014). In animals, the stimulatory effect of Ang II on the respiratory system may be reciprocally inhibited by AVP in a V1aR-dependent manner (Anderson et al., 1990; Walker and Jennings, 1994, 1995), which may involve inhibition of the renin release (Bunag et al., 1967).

## Vasopressin/Copeptin Release Under Pathological Conditions

Next, we briefly summarize clinical observations indicating that AVP is released in excess under conditions of pulmonary dysfunction and medical conditions associated with abnormal breathing patterns. This raises a possibility that AVP is not only a biomarker of respiratory disturbances and hypoxia, but that it may also contribute to the regulation of the respiratory system.

### Respiratory Disorders

Increased plasma concentration of copeptin, which reflects the increase in AVP release, is observed in several respiratory disturbances (Szczepanska-Sadowska et al., 2017). It is present in chronic obstructive pulmonary disease (COPD), in which copeptin level is predictive for the recurrence of exacerbation and all-cause mortality (Muller et al., 2007; Zhao et al., 2014). Increased plasma copeptin level is also present in patients with lower respiratory tract infection, especially in patients with community acquired pneumonia, in whom it has a predictive value for all-cause mortality, clinical instability, and deterioration (Muller et al., 2007; Kruger et al., 2010; Kolditz et al., 2012). High copeptin concentration is also present in acute respiratory distress syndrome, acute lung injury, and cardiopulmonary oedema, where elevated copeptin has a better predictive value for short-term mortality than NT-proBNP, a marker of cardiac ventricles’ overload (Lin et al., 2012). Finally, some studies also indicate the potential role of copeptin in the diagnosis and risk stratification of patients with pulmonary hypertension (Nickel et al., 2013) and pulmonary embolisms (Hellenkamp et al., 2018). Recently, it has been shown that elevated concentrations of copeptin are associated with worse outcome in COVID-19 patients with SARS-CoV-2 infection (Gregoriano et al., 2021) and may discriminate between patients with COVID-19 and patients with community-acquired pneumonia (Kuluöztürk et al., 2021).

### Non-respiratory Disorders

Besides disturbances of the respiratory system, elevated plasma concentrations of AVP or copeptin are found in several non-respiratory disorders that are often accompanied by abnormal respiratory patterns and increased ventilation. Specifically, elevated copeptin concentration is predictive of worse outcome in patients with traumatic brain injury (Yang et al., 2014; Zhang et al., 2014; Choi et al., 2017), a condition associated with increased expression of V1aRs in the brain (Szmydynger-Chodobska et al., 2004) and abnormal respiratory patterns (Racca et al., 2020). They are also seen in children with febrile and epileptic seizures (Evers et al., 2020). Furthermore, increased plasma concentration of copeptin has been found in patients with acute and chronic heart failure (Maisel et al., 2011; Düngen et al., 2018; Schill et al., 2021), a condition usually accompanied by abnormal respiratory patterns, such as Cheyne-Stokes breathing and sleep apnoea (Cowie et al., 2021). Increase copeptin levels are also found in patients admitted to intensive care unit or emergency department with acute severe dyspnoea due to non-respiratory causes and accompanied by hypoxia and increased respiratory rate (Ara-Somohano et al., 2017).

## The Respiratory Effects of Vasopressin

Vasopressin affects the respiratory system both as a neurohormone and as a neurotransmitter. Available evidence indicates that its respiratory effects are mainly mediated by V1aRs. Vasopressin present in the bloodstream may affect the respiratory system by interacting with V1aRs expressed in the lungs (Tahara et al., 1998), the circumventricular organs (CVOs; Raggenbass et al., 1989; Ostrowski et al., 1994; Tribollet et al., 1999; Hindmarch et al., 2011), and in the carotid bodies (Żera et al., 2018). In addition to circulating AVP, vasopressinergic neurons projecting to the brainstem respiratory centers affect respiratory activity *via* V1aRs (Kc et al., 2002a, 2010; Chuang et al., 2005; Kc and Dick, 2010). It appears that the effects of AVP on the respiratory function is site-specific and may result in opposing effects, as discussed below.

### Vasopressin Receptors in the Respiratory System, Chemoreceptors and Respiratory Centers of the Brain

#### Lungs and Pulmonary Circulation

It has been shown that V1aRs are predominantly expressed in the lung (Hirasawa et al., 1994; Tahara et al., 1998) and in the pulmonary arteries (Enomoto et al., 2014). Contrary to arteries in the systemic circulation, AVP does not cause vasoconstriction of the pulmonary arteries, but rather it causes vasodilation, especially under conditions of hypoxic vasoconstriction of pulmonary vessels in animals (Walker et al., 1989; Wallace et al., 1989; Enomoto et al., 2014; Sugawara et al., 2019) and humans (Jeon et al., 2006; Currigan et al., 2014). Thus, AVP does not increase pulmonary vascular resistance and the right ventricle’s afterload, but it only selectively increases the vascular resistance in systemic circulation (Walker et al., 1989; Jeon et al., 2006).

Retrograde transneuronal labeling indicates that the airway-related vagal preganglionic neurons receive innervation from vasopressinergic neurons of the PVN (Kc et al., 2006, 2010). Furthermore, AVP acting on V1aRs depolarizes and increases firing rate of these preganglionic neurons (Yan et al., 2017), which is suggestive of the broncho-constrictive and secretory effect of AVP. Nonetheless, available evidence points to limited effects of AVP on bronchoconstriction in laboratory animals (Bhoola et al., 1962; Zheng et al., 2017) and humans (Knox et al., 1989).

#### Arterial Chemoreceptors

Recently, we showed presence of immunostaining for both C-terminal and N-terminal fragments of the V1aR in the chemosensitive glomus cells of the carotid bodies collected from the normotensive Sprague-Dawley rats (Żera et al., 2018). Furthermore, expression of G protein q/11 and phosphokinase C, key intracellular components of the V1aR signaling, has been detected in the chemosensitive glomus cells with the single-cell transcriptomics in mouse (Zhou et al., 2016). These findings indicate that circulating AVP may affect the glomus cells expressing V1aRs and presumably modulate the activity of the carotid body.

#### Circumventricular Organs

Circumventricular organs (CVOs) include the organum vasculosum laminae terminalis (OVLT) and the SFO that are located close to the third cerebral ventricle, and the area postrema (AP), which is situated in the dorsal surface of the medulla oblongata. They express numerous receptors, lack the blood-brain barrier and are accessible for hormones and mediators circulating in the bloodstream (Ufnal and Skrzypecki, 2014). All CVOs express receptors for AVP, predominantly V1aRs (Raggenbass et al., 1989; Jurzak and Schmid, 1998; McKinley et al., 1999; Tribollet et al., 1999; Kc and Dick, 2010; Hindmarch et al., 2011). Upon binding to V1aRs in the CVOs of the third ventricle, AVP exerts sympathoexcitatory and pressor effects (Ufnal and Skrzypecki, 2014; Szczepanska-Sadowska et al., 2017; Japundžić-Žigon et al., 2020). In contrast, stimulation of V1aRs in the AP sensitizes the arterial baroreflex and results in hypotension (Hasser and Bishop, 1990; Japundžić-Žigon et al., 2020), which indicates opposite effects of AVP depending on the place of its action. A limited number of studies indicates that administration of AVP into the cerebral ventricles or directly into the AP decreases respiratory rate and phrenic nerve activity (Zerbe and Feuerstein, 1985; Yang et al., 2006).

#### Respiratory Centers of the Brain

In rats, V1aRs are expressed on the sympathetic neurons of the rostral ventrolateral medulla (RVLM) and the respiratory neurons located in the rostral ventral respiratory column (rVRC; Kc and Dick, 2010) and pre-Bötzinger complex (Kc et al., 2002a). Retrograde labeling studies show that in addition to these nuclei, the phrenic nuclei receive vasopressinergic innervation from the PVN (Kc et al., 2002a,b). The expression of V1aRs in these brainstem structures is augmented by exposure of the animals to hypoxia (Kc and Dick, 2010; Kc et al., 2010). Furthermore, a significant subpopulation of the vasopressinergic PVN neurons are activated by hypercapnia (Kc et al., 2002b). Importantly, binding of AVP to V1 receptors was found in neurons across the nucleus of the solitary tract (NTS), including its caudal part (Raggenbass et al., 1989) that receives sensory input from the arterial chemoreceptors (Lipski et al., 1976, 1977; Finley and Katz, 1992; Zera et al., 2019).

### Respiratory Effects of Vasopressin Acting as a Hormone

Circulating AVP may affect the neural control of the respiratory system either by binding to its receptors in the CVOs or receptors located in other peripheral tissues, especially the arterial chemoreceptors (Figure 1). Overall, prevailing evidence indicates that AVP as a hormone present in the bloodstream supresses ventilation. We hypothesize that under conditions of hypoxia, oligo/hypovolemia, hypotonia, hypoglycemia, exercise, activation of renin-angiotensin system, the increase in AVP plasma concentration may help in limiting excessive increase in ventilation and prevent development of hypocapnia. Thus, it is likely that inhibition of ventilation by AVP may provide a fine-tuning mechanism that maintains respiratory activity at the most efficient level.

#### Vasopressin Circulating in the Bloodstream

Intravenous infusions of AVP transiently decrease pulmonary ventilation and phrenic nerve activity in conscious dogs (Ohtake and Jennings, 1993), anesthetized and awake rats (Louwerse and Marshall, 1993; Walker and Jennings, 1998; Żera et al., 2018; Brackley and Toney, 2021) and fetal lambs (Bessho et al., 1997). Recently published pilot study in patients with autosomal dominant polycystic kidney disease showed that upon initiation of treatment with tolvaptan, a selective V2R antagonist, plasma copeptin level increased 6-fold and this was associated with a modest but significant increase in arterial carbon-dioxide and plasma acidity (Heida et al., 2021), suggestive of ventilatory inhibition by increased AVP levels in the bloodstream and enhanced stimulation of V1 receptors, which were not blocked by tolvaptan.

The inhibitory effects of AVP on the respiratory activity depend on V1aRs, as blockade of these receptors with selective antagonists completely prevents changes in pulmonary ventilation induced by systemic administration of AVP in anesthetized rats (Louwerse and Marshall, 1993; Żera et al., 2018; Brackley and Toney, 2021). Furthermore, administration of V1aR antagonists may result in the increase in pulmonary ventilation in awake dogs (Walker and Jennings, 1994). However, it should be noted that insignificant effect of V1aR blockade on pulmonary ventilation was seen in conscious rats (Walker and Jennings, 1998). Furthermore, intravenous administration of V1aR peptidergic antagonist, that does not cross the blood-brain barrier, has insignificant effects on hypoxia-induced increase in the pulmonary ventilation in conscious dogs (Overgaard et al., 1996), suggesting lack of involvement of V1aRs located in the peripheral organs or CVOs in the increase in ventilation evoked by activation of the arterial chemoreflex in this species. However, systemic administration of V1aR antagonist unmasks stimulatory effects of Ang II on the respiration in dogs (Walker and Jennings, 1995). Interestingly, oxytocin, which is structurally related to AVP and acts as a weak agonist of the V1aRs (Szczepanska-Sadowska et al., 2017), is capable of reversing the opioid-induced respiratory depression and this effect is fully unmasked by blockade of V1aRs (Brackley and Toney, 2021).

One of the plausible explanations of the inhibitory action of systemic administration of AVP on ventilation is a baroreflex-mediated inhibition of the medullary respiratory neurons (Richter and Seller, 1975; Brunner et al., 1982; Baekey et al., 2010; McMullan and Pilowsky, 2010) due to the AVP-mediated rise in arterial blood pressure. However, as mentioned above the arterial baroreflex-mediated inhibition of the pulmonary ventilation in the conscious rat is vaguely pronounced (Walker and Jennings, 1996). Thus, another mechanism is presumably at play.

#### Vasopressin and the Circumventricular Organs

Based on the experimental work, Jennings (1994) suggested that angiotensin II and AVP present in the bloodstream may affect ventilation *via* interaction with the CVOs. Thus, one of such mechanisms may involve stimulation of the V1aRs in the CVOs, specifically in the AP. In addition to the AP neurons responding to both AVP and the rise in blood pressure, indicating their pressure-sensitivity, there are at least two pools of neurons in the AP that respond to circulating AVP independently from the changes in arterial blood pressure, indicating their AVP-sensitivity. One pool increases and another one decreases the firing rate in response to intravenous AVP (Smith et al., 1994). Both types of responses are dependent on vasopressin V1 receptors, as their inhibition abolishes AVP-induced changes in the discharge frequency (Smith et al., 1994). Furthermore, in the medial regions of the NTS there are neurons that respond with increased or decreased firing in response to AVP microinjections into the AP (Cai et al., 1994), indicating that the circulating neurohormone may affect activity of the NTS involved in the baro- and chemoreflexes *via* the neuronal pathway including the AP. In fact, application of AVP into the AP significantly reduces the phrenic nerve activity in mechanically ventilated, urethane anesthetized rats, and the inhibitory effect is prevented by local administration of V1aR antagonist into the AP or by pharmacological inhibition of the NTS (Yang et al., 2006). This is further supported by observations that AVP administered into the cerebral ventricles, thus allowing for interaction of the neurohormone with the CVOs, decreases respiratory rate in the anesthetized rat (Zerbe and Feuerstein, 1985). However, it should be noted that intracerebroventricular infusion of AVP in conscious macaque monkey had no effect on ventilatory parameters (Lee et al., 1985). This is contrary to the studies with electrical and pharmacological stimulation of the AP neurons in rabbits, which results in increased phrenic nerve activity (Srinivasan et al., 1993; Bongianni et al., 1994). It should be noted that electrical stimulation of the AP may trigger both excitation or inhibition of the NTS neurons in rodents (Chrobok et al., 2021). Similarly, electric stimulation of the SFO increases ventilation in rats (Ferguson et al., 1989). Although, the V1aRs are expressed in the SFO (Ostrowski et al., 1994), thus far it has not been determined how their stimulation affects the respiratory system. Available evidence and anatomical proximity to the respiratory neurons of the brainstem point to the AP as a putative CVO involved in mediating the respiratory effects of circulating AVP. However, given the limited number of studies that evaluated respiratory responses to AVP directly administered into the CVO, the role of CVOs in neural control of respiration by AVP awaits further corroboration from experiments specifically targeting AVP and the AP and/or the SFO, especially under conditions associated with activation of vasopressinergic system and in awake animals.

#### Vasopressin and the Carotid Bodies

Circulating AVP may also affect control of the respiratory system by binding to its receptors within the carotid body. We showed that in normotensive rats, AVP administered locally into the carotid body *via* the internal carotid artery causes a modest increase in the pulmonary ventilation without significant changes in the blood pressure (Żera et al., 2018). Further studies are needed to determine whether these effects are directly dependent on activation of the chemosensitive glomus cells that express V1aRs (Żera et al., 2018) or rather on AVP-mediated decrease in the carotid body blood flow that may sensitize the chemoreceptors (Przybylski, 1981; Brognara et al., 2021).

### Respiratory Effects of Vasopressin Acting as a Neurotransmitter in the Central Nervous System

The paraventricular nucleus of the hypothalamus is the main source of the vasopressinergic innervation of the brainstem and provides projections to the discrete nuclei of the brainstem and the spinal cord (Coote, 1995; Pyner, 2009; Coote and Spyer, 2018). Available evidence indicates that these vasopressinergic pathways may participate in the regulation of breathing.

#### Vasopressin and the Paraventricular Nucleus

Retrograde labeling and functional studies show neural pathways between the PVN and the NTS, the RVLM, and the presympathetic neurons of the spinal cord (Yang and Coote, 1998; Yang et al., 2002; Ferguson et al., 2008; Affleck et al., 2012). The PVN is involved in the peripheral chemoreflex, as the inhibition or disinhibition of the PVN neurons result in the respective attenuation or augmentation of the peripheral chemoreflex-evoked sympathetic and respiratory responses (Reddy et al., 2005). Furthermore, retrograde labeling studies combined with immunostaining for V1aRs show that the PVN vasopressinergic fibers terminate on neurons in the RVLM, the rVRC, the pre-Bötzinger complex, the NTS, and the phrenic nuclei (Kc et al., 2002a, 2010; Jackson et al., 2005; Figure 1). In a series of experiments, Kc et al. (2002b) showed that hypercapnia activates vasopressinergic neurons in the PVN and hypoxia upregulates V1aRs in the RVLM, the ventral respiratory group and in the phrenic nuclei in the spinal cord (Kc et al., 2010). They also showed that in anesthetized mechanically ventilated and vagotomized rats, disinhibition of the PVN leads to increase in respiratory activity, as estimated by means of electromyography of the diaphragm and genioglossal muscle and that this increase can be prevented by a pre-treatment of the RVLM and the rVRC with selective V1aR antagonist (Kc et al., 2010). Along with the upregulation of V1aRs, the respiratory responses were augmented by chronic intermittent hypoxia (Kc et al., 2010). The increases in respiratory activity evoked by disinhibition of the PVN were accompanied by pressor response and dependent on V1aRs (Kc and Dick, 2010, Kc et al., 2010). Together, these studies indicate that activation of the vasopressinergic PVN projections to the RVLM/rVRC increases the respiratory activity *via* V1aRs.

#### Vasopressin and the Ventral Lateral Medulla

In addition, microinjections of AVP into the pre-Bötzinger complex or RVLM/rVRC also increased the diaphragm’s muscle activity in a V1aR-dependent manner (Kc et al., 2002a, 2010). The increases in respiratory activity evoked by local application of AVP was accompanied by pressor response (Kc et al., 2002a). Furthermore, microinjection of the V1aR antagonist into the RVLM/rVRC resulted in a decrease in respiratory activity in rats exposed to chronic intermittent hypoxia (Kc et al., 2010), suggestive of tonic vasopressinergic input to the respiratory neurons. Together, these studies indicate that AVP and V1aRs in the RVLM and rVRC are involved in stimulation of the respiratory activity.

Contrary effects were reported in a series of experiments, in which AVP was microinjected into various sites of the ventrolateral medulla (VLM) caudal from the RVLM determined as a pressor region of the VLM in urethane anesthetized and vagotomized rats that were paralyzed and mechanically ventilated. In these experiments, microinjections of AVP applied into the VLM and the rostral ventral respiratory group (rVRG) caudal from the RVLM resulted in apnoea with subsequent inhibition of the phrenic nerve discharges (Chuang et al., 2003, 2005). Although, the inhibitory effect of AVP on the respiratory activity was consistent, the pressor response was dependent on the site of injection. Applications of AVP into the lateral part of the VLM decreased the phrenic nerve activity and concomitantly elevated arterial blood pressure, whereas AVP application into the medial part of the VLM attenuated only the phrenic nerve activity, without significant effect on the arterial blood pressure (Chuang et al., 2003, 2005; Cheng et al., 2004). Microinjections of AVP into the VLM and the rVRG were also shown to inhibit activity of the hypoglossal nerve (Chuang et al., 2005). Of note, these inhibitory effects of AVP on phrenic nerve activity were diminished by hypercapnia (Chuang et al., 2003; Cheng et al., 2004) and were completely abolished by pre-treatment with a selective V1aR antagonist (Chuang et al., 2003, 2005; Cheng et al., 2004). Administration of the V1aR antagonist under resting conditions had insignificant effect on blood pressure and phrenic nerve activity in these experiments (Chuang et al., 2003, 2005; Cheng et al., 2004), suggesting lack of significant impact of vasopressinergic efferents innervating these structures on ventilation at rest. Together, these findings suggest inhibitory action of AVP on the respiratory neurons of the rVRG located caudally from the RVLM.

These two series of reports provide apparently conflicting results that may be caused by the several differences in the experimental paradigms, including use of neuromuscular blockade and atropine. In addition, mechanical ventilation with continuous positive end-expiratory pressure potently increases plasma concentration of AVP (Annat et al., 1983; Venus et al., 1985) and this activation of AVP release depends on the hydration status of the ventilated animal (Venus et al., 1985). Thus, various levels of baseline activity of vasopressinergic system could have been present across the above studies. It should be also noted that acute experiments under anesthesia investigating the autonomic responses related to the function of the brainstem and the hypothalamus may yield conflicting results to physiological responses observed in conscious animals (Kannan et al., 1989). Nonetheless, it seems that in the more rostral part of the VLM/rVRC AVP promotes respiratory activity, whereas in the more caudal area, it inhibits respiration.

#### Vasopressin and the Nucleus of the Solitary Tract

The carotid body-evoked hyperglycemic response has been recognized as a contributing factor in the pathophysiology of metabolic syndrome (Conde et al., 2017) and blockade of V1aRs in the NTS was shown to attenuate increase in the plasma concentration of glucose induced by activation of the peripheral chemoreflex (Montero et al., 2006). Moreover, inhibition of the V1aRs in the NTS substantially reduces pressor and tachycardic effects of electrical stimulation of the PVN (Pittman and Franklin, 1985). Thus, it is probable that AVP in the NTS also modulates respiratory reflexes, especially those originating from the peripheral chemoreceptors. This assumption is further substantiated by evidence indicating that almost half of the NTS neurons respond with excitation to local application of AVP in the coronal sections of the rat’s brainstem (Raggenbass et al., 1989). Furthermore, in anesthetized cat, iontophoretic applications of AVP into the NTS revealed excitatory effects of the neuropeptide on the inspiratory neurons of the NTS (Henry and Sessle, 1989). However, microinjections of AVP into a discrete region of the NTS rostral to the calamus scriptorius also produced inhibition of the phrenic nerve activity in rats (Yang et al., 2006). Further investigations are required to elucidate the significance of AVP action in the NTS for regulation of respiration.

The role of endogenous AVP and vasopressinergic neurons in the regulation of the respiratory system is also indirectly supported by spectral analysis of the blood pressure variability, the heart rate variability, and selective pharmacological inhibition of AVP receptors in the central nervous system in awake rats, which suggests that vasopressin and V1aR/V1bRs are involved in enhancing ventilation and respiratory-induced blood pressure oscillations (Japundzic-Zigon, 2001; Milutinović et al., 2006). Furthermore, experiments in the Brattleboro rats lacking AVP indicate that although under resting conditions their breathing is normal, under challenging conditions of septic shock their respiratory adaptation, i.e., increase in respiration, is absent (Brackett et al., 1983), which suggests the important role of the neuropeptide in respiratory adaptation to disturbed homeostasis. This is further supported by known respiratory problems, including sleep apnoea, decreased ventilation and spells in patients with Wolfram syndrome characterized by diabetes insipidus and a lack of AVP (Thompson et al., 1989; Licis et al., 2019).

## Future Perspectives

Evidence pointing to the involvement of AVP in the regulation of respiration mainly emerges from experimental studies in anesthetized animals, in which either local or systemic injections of the neuropeptide or its selective antagonists were performed. Given the complexity of AVP-induced responses including water-electrolyte, cardiovascular, metabolic and behavioral ones, novel approaches are needed to decipher the integrative role of AVP in the regulation of breathing in conscious and intact animals under physiological conditions and in disturbed homeostasis.

Targeted control and modification of the vasopressinergic neurons with optogenetics and chemogenetics have been proposed for determining the role of AVP in the regulation of physiological functions and behavior (Murphy et al., 2012; Yoshimura and Ueta, 2019). These novel techniques open prospects for dissecting vasopressinergic pathways and functional role of AVP in the regulation of the respiratory system. Recently, chemogenetic activation of endogenous AVP has been applied in figuring out the anorexigenic effects of AVP (Yoshimura et al., 2017; Sanada et al., 2021). Targeted optogenetic stimulation/inhibition of vasopressinergic neurons in the hypothalamus and extra-hypothalamic regions or vasopressinergic efferents innervating specific brain structures have been used in various experimental models (Ishii et al., 2016; Smith et al., 2016; Hume et al., 2019; Yoshimura and Ueta, 2019; Tabarean, 2021)., Moreover, successful optogenetic stimulation or inhibition of the SFO or the OVLT neurons revealed novel information on the regulation of thirst and drinking, vasopressinergic neurons and control of blood pressure (Oka et al., 2015; Zimmerman et al., 2016; Pool et al., 2020; Frazier et al., 2021). The optogenetic approach has been also proposed (Abdala et al., 2015) and successfully applied in delineating respiratory networks of the brainstem (Alsahafi et al., 2015; Basting et al., 2015; Koizumi et al., 2016; Cregg et al., 2017; Malheiros-Lima et al., 2018; Fortuna et al., 2019; Ikeda et al., 2019; Souza et al., 2020). These new technologies may help in dissecting the role of AVP in regulation of breathing by precise chemogenetic or optogenetic activation of vasopressinergic neurons projecting to the brainstem, optogenetic inhibition or stimulation of specific pools of respiratory neurons within the brainstem and CVOs, especially ones with V1aR phenotype, and targeted modulation of vasopressinergic fibers supplying them. Such experimental manipulations carried out in awake animals in physiological state and under conditions of hypo/hyperosmolality, hypovolemia, hypotension, hypoglycemia, hypoxia, exercise, or activation of renin-angiotensin system will bring to light the integrative role of AVP acting both as a neurohormone and a neurotransmitter in adjusting the respiratory system to disturbed homeostasis.

## Conclusion

Vasopressin is one of the key hormones involved in maintaining body homeostasis and plays critical role in adjusting various body systems to changing internal and external environments. A great attention has been dedicated the role of AVP in maintaining renal water-electrolyte balance and regulation of the circulatory system. Recently, the central effects of AVP related to cognition, mood, memory, and pain have been recognized. Available evidence indicates that AVP also plays a complex role in the regulation of the respiratory system. As a neurohormone present in the circulation, AVP influences the respiratory activity *via* the circumventricular organs, especially the AP, and the arterial chemoreceptors, specifically those located in the carotid bodies. In the area postrema, AVP inhibits the phrenic nerve activity, whereas in the carotid bodies it appears to promote ventilation. Acting as a neurotransmitter in the brainstem, AVP exerts both stimulatory and inhibitory effects on the phrenic nerve activity in a site-specific manner; however, this awaits further investigations in conscious animals. The respiratory effects of AVP appear to be predominantly mediated by the vasopressin V1a receptors. Strong evidence indicates that vasopressin may be an important neuropeptide involved in maintaining respiratory homeostasis.

## Author Contributions

MP designed the study, prepared the figure, and drafted the initial version of the manuscript. JP designed the study and revised the manuscript. AC-J and ES-S revised the manuscript. TŻ conceived the idea of the review, designed the study, prepared the figure, and revised and edited the final version of the manuscript. All authors contributed to the article and approved the submitted version.

## Funding

The study was funded by Diamond Grant from the Polish Ministry of Education and Science, grant number DI2018 020648, awarded to MP. The study was carried out with the use of CePT infrastructure financed by the European Union – the European Regional Development Fund within the Operational Programme “Innovative economy” for 2007–2013.

## Conflict of Interest

The authors declare that the research was conducted in the absence of any commercial or financial relationships that could be construed as a potential conflict of interest.

## Publisher’s Note

All claims expressed in this article are solely those of the authors and do not necessarily represent those of their affiliated organizations, or those of the publisher, the editors and the reviewers. Any product that may be evaluated in this article, or claim that may be made by its manufacturer, is not guaranteed or endorsed by the publisher.

## References

[ref1] Abdala A. P. Paton J. F. R. Smith J. C. (2015). Defining inhibitory neurone function in respiratory circuits: opportunities with optogenetics? J. Physiol. 593, 3033–3046. doi: 10.1113/jphysiol.2014.280610, PMID: 25384785 PMC4532524

[ref2] Affleck V. S. Coote J. H. Pyner S. (2012). The projection and synaptic organisation of NTS afferent connections with presympathetic neurons, GABA and nNOS neurons in the paraventricular nucleus of the hypothalamus. Neuroscience 219, 48–61. doi: 10.1016/j.neuroscience.2012.05.070, PMID: 22698695 PMC3409377

[ref3] Aguilera G. Rabadan-Diehl C. (2000). Vasopressinergic regulation of the hypothalamic-pituitary-adrenal axis: implications for stress adaptation. Regul. Pept. 96, 23–29. doi: 10.1016/s0167-0115(00)00196-8, PMID: 11102648

[ref4] Aguilera G. Subburaju S. Young S. Chen J. (2008). The parvocellular vasopressinergic system and responsiveness of the hypothalamic pituitary adrenal axis during chronic stress. Prog. Brain Res. 170, 29–39. doi: 10.1016/S0079-6123(08)00403-2, PMID: 18655869 PMC2536760

[ref5] Alsahafi Z. Dickson C. T. Pagliardini S. (2015). Optogenetic excitation of preBötzinger complex neurons potently drives inspiratory activity in vivo. J. Physiol. 593, 3673–3692. doi: 10.1113/JP270471, PMID: 26010654 PMC4560590

[ref6] Anderson J. W. Sarda I. R. Jennings D. B. (1990). Acute changes in osmolality and renin and respiratory control of arterial PCO2 and [H+]. Respir. Physiol. 80, 1–16. doi: 10.1016/0034-5687(90)90002-G, PMID: 2114660

[ref7] Anderson J. W. Smith P. M. Ferguson A. V. (2001). Subfornical organ neurons projecting to paraventricular nucleus: whole-cell properties. Brain Res. 921, 78–85. doi: 10.1016/S0006-8993(01)03093-1, PMID: 11720713

[ref8] Anderson J. W. Washburn D. L. Ferguson A. V. (2000). Intrinsic osmosensitivity of subfornical organ neurons. Neuroscience 100, 539–547. doi: 10.1016/S0306-4522(00)00313-4, PMID: 11098117

[ref9] Annat G. Viale J. P. Bui Xuan B. Hadj Aissa O. Benzoni D. Vincent M.. (1983). Effect of PEEP ventilation on renal function, plasma renin, aldosterone, neurophysins and urinary ADH, and prostaglandins. Anesthesiology 58, 136–141. doi: 10.1097/00000542-198302000-00006, PMID: 6337527

[ref10] Aoki R. Yokoyama U. Ichikawa Y. Taguri M. Kumagaya S. Ishiwata R.. (2014). Decreased serum osmolality promotes ductus arteriosus constriction. Cardiovasc. Res. 104, 326–336. doi: 10.1093/cvr/cvu199, PMID: 25190043

[ref11] Ara-Somohano C. Bonadona A. Carpentier F. Pavese P. Vesin A. Hamidfar-Roy R.. (2017). Evaluation of eight biomarkers to predict short-term mortality in patients with acute severe dyspnea. Minerva Anestesiol. 83, 824–835. doi: 10.23736/S0375-9393.17.10882-5, PMID: 28275223

[ref12] Arnauld E. Czernichow P. Fumoux F. Vincent J. D. (1977). The effects of hypotension and hypovolaemia on the liberation of vasopressin during haemorrhage in the unanaesthetized monkey (Macaca mulatta). Pflugers Arch. 371, 193–200. doi: 10.1007/BF00586258, PMID: 414200

[ref13] Baekey D. M. Molkov Y. I. Paton J. F. R. Rybak I. A. Dick T. E. (2010). Effect of baroreceptor stimulation on the respiratory pattern: insights into respiratory-sympathetic interactions. Respir. Physiol. Neurobiol. 174, 135–145. doi: 10.1016/j.resp.2010.09.006, PMID: 20837166 PMC3691868

[ref14] Baker M. A. Dawson D. D. (1985). Inhibition of thermal panting by intracarotid infusion of hypertonic saline in dogs. Am. J. Physiol. 249, R787–R791. doi: 10.1152/ajpregu.1985.249.6.R787, PMID: 4073298

[ref15] Bankir L. Bichet D. G. Morgenthaler N. G. (2017). Vasopressin: physiology, assessment and osmosensation. J. Intern. Med. 282, 284–297. doi: 10.1111/joim.12645, PMID: 28649750

[ref16] Basting T. M. Burke P. G. R. Kanbar R. Viar K. E. Stornetta D. S. Stornetta R. L.. (2015). Hypoxia silences retrotrapezoid nucleus respiratory chemoreceptors via alkalosis. J. Neurosci. 35, 527–543. doi: 10.1523/JNEUROSCI.2923-14.2015, PMID: 25589748 PMC4293409

[ref17] Baylis P. H. (1987). Osmoregulation and control of vasopressin secretion in healthy humans. Am. J. Physiol. 253, R671–R678. doi: 10.1152/ajpregu.1987.253.5.R671, PMID: 3318505

[ref18] Baylis P. H. Robertson G. L. (1980). Plasma vasopressin response to hypertonic saline infusion to assess posterior pituitary function. J. R. Soc. Med. 73, 255–260. doi: 10.1177/014107688007300408, PMID: 7241442 PMC1437414

[ref19] Baylis P. H. Zerbe R. L. Robertson G. L. (1981). Arginine vasopressin response to insulin-induced hypoglycemia in man. J. Clin. Endocrinol. Metab. 53, 935–940. doi: 10.1210/jcem-53-5-935, PMID: 7026596

[ref20] Berkenbosch F. De Goeij D. C. Tilders F. J. (1989). Hypoglycemia enhances turnover of corticotropin-releasing factor and of vasopressin in the zona externa of the rat median eminence. Endocrinology 125, 28–34. doi: 10.1210/endo-125-1-28, PMID: 2544403

[ref21] Bessho T. Murata Y. Ninomiya Y. Ibara S. Yamamoto T. Miyake Y.. (1997). Effect of arginine vasopressin on breathing movements of chronically instrumented fetal lambs. Acta Obstet. Gynecol. Scand. 76, 107–111. doi: 10.3109/00016349709050063, PMID: 9049280

[ref22] Bhoola K. D. Collier H. O. Schachter M. Shorley P. G. (1962). Actions of some peptides on bronchial muscle. Br. J. Pharmacol. Chemother. 19, 190–197. doi: 10.1111/j.1476-5381.1962.tb01439.x, PMID: 13868845 PMC1482229

[ref23] Bichet D. G. (2019). Regulation of thirst and vasopressin release. Annu. Rev. Physiol. 81, 359–373. doi: 10.1146/annurev-physiol-020518-114556, PMID: 30742785

[ref24] Bin-Jaliah I. Maskell P. D. Kumar P. (2004). Indirect sensing of insulin-induced hypoglycaemia by the carotid body in the rat. J. Physiol. 556, 255–266. doi: 10.1113/jphysiol.2003.058321, PMID: 14742728 PMC1664881

[ref25] Bongianni F. Mutolo D. Srinivasan M. Staderini G. Baccari M. C. Calamai F.. (1994). Gastric relaxation in response to chemical stimulation of the area postrema in the rabbit. Brain Res. 646, 307–311. doi: 10.1016/0006-8993(94)90095-7, PMID: 8069679

[ref26] Brackett D. J. Schaefer C. F. Wilson M. F. (1983). The role of vasopressin in the maintenance of cardiovascular function during early endotoxin shock. Adv. Shock Res. 9, 147–156. PMID: 6880966

[ref27] Brackley A. D. Toney G. M. (2021). Oxytocin receptor activation rescues opioid-induced respiratory depression by systemic fentanyl in the rat. J. Pharmacol. Exp. Ther. 378, 96–107. doi: 10.1124/jpet.121.000535, PMID: 33990416 PMC8407530

[ref28] Bristow J. D. Brown E. B. J. Cunningham D. J. Goode R. C. Howson M. G. Sleight P. (1971). The effects of hypercapnia, hypoxia and ventilation on the baroreflex regulation of the pulse interval. J. Physiol. 216, 281–302. doi: 10.1113/jphysiol.1971.sp009525, PMID: 4326995 PMC1331939

[ref29] Brognara F. Felippe I. S. A. Salgado H. C. Paton J. F. R. (2021). Autonomic innervation of the carotid body as a determinant of its sensitivity: implications for cardiovascular physiology and pathology. Cardiovasc. Res. 117, 1015–1032. doi: 10.1093/cvr/cvaa250, PMID: 32832979

[ref30] Brunner M. J. Sussman M. S. Greene A. S. Kallman C. H. Shoukas A. A. (1982). Carotid sinus baroreceptor reflex control of respiration. Circ. Res. 51, 624–636. doi: 10.1161/01.RES.51.5.624, PMID: 7139881

[ref31] Buijs R. M. (1978). Intra- and extrahypothalamic vasopressin and oxytocin pathways in the rat. Pathways to the limbic system, medulla oblongata and spinal cord. Cell Tissue Res. 192, 423–435. doi: 10.1007/BF00212323, PMID: 699026

[ref32] Bunag R. D. Page I. H. McCubbin J. W. (1967). Inhibition of renin release by vasopressin and angiotensin*. Cardiovasc. Res. 1, 67–73. doi: 10.1093/cvr/1.1.67, PMID: 4293924

[ref33] Cai Y. Hay M. Bishop V. S. (1994). Stimulation of area postrema by vasopressin and angiotensin II modulates neuronal activity in the nucleus tractus solitarius. Brain Res. 647, 242–248. doi: 10.1016/0006-8993(94)91323-4, PMID: 7922500

[ref34] Caraty A. Grino M. Locatelli A. Guillaume V. Boudouresque F. Conte-Devolx B.. (1990). Insulin-induced hypoglycemia stimulates corticotropin-releasing factor and arginine vasopressin secretion into hypophysial portal blood of conscious, unrestrained rams. J. Clin. Invest. 85, 1716–1721. doi: 10.1172/JCI114626, PMID: 2161426 PMC296631

[ref35] Cheng M.-T. Chuang C.-W. Lin J.-T. Hwang J.-C. (2004). Cardiopulmonary response to vasopressin-induced activation on V1A receptors in the lateral ventrolateral medulla in the rat. Chin. J. Phys. 47, 31–42. PMID: 15239592

[ref36] Chiodera P. Volpi R. Capretti L. Speroni G. Marcato A. Rossi G.. (1992). Hypoglycemia-induced arginine vasopressin and oxytocin release is mediated by glucoreceptors located inside the blood-brain barrier. Neuroendocrinology 55, 655–659. doi: 10.1159/000126185, PMID: 1321355

[ref37] Choi K.-S. Cho Y. Jang B.-H. Kim W. Ahn C. Lim T. H.. (2017). Prognostic role of copeptin after traumatic brain injury: a systematic review and meta-analysis of observational studies. Am. J. Emerg. Med. 35, 1444–1450. doi: 10.1016/j.ajem.2017.04.038, PMID: 28545954

[ref38] Chrobok L. Wojcik M. Klich J. D. Pradel K. Lewandowski M. H. Piggins H. D. (2021). Phasic neuronal firing in the rodent nucleus of the solitary tract ex vivo. Front. Physiol. 12:638695. doi: 10.3389/fphys.2021.638695, PMID: 33762969 PMC7982836

[ref39] Chuang C.-W. Cheng M.-T. Lin J.-T. Hsien H.-Y. Hung H.-Y. Hwang J.-C. (2003). Arginine vasopressin produces inhibition upon respiration without pressor effect in the rat. Chin. J. Phys. 46, 71–81. PMID: 12974298

[ref40] Chuang C.-W. Cheng M.-T. Yang S.-J. Hwang J.-C. (2005). Activation of ventrolateral medulla neurons by arginine vasopressin via V1A receptors produces inhibition on respiratory-related hypoglossal nerve discharge in the rat. Chin. J. Phys. 48, 144–154. PMID: 16304841

[ref41] Colucci G. Stutz M. Rochat S. Conte T. Pavicic M. Reusser M.. (2014). The effect of desmopressin on platelet function: a selective enhancement of procoagulant COAT platelets in patients with primary platelet function defects. Blood 123, 1905–1916. doi: 10.1182/blood-2013-04-497123, PMID: 24443440

[ref42] Conde S. V. Ribeiro M. J. Melo B. F. Guarino M. P. Sacramento J. F. (2017). Insulin resistance: a new consequence of altered carotid body chemoreflex? J. Physiol. 595, 31–41. doi: 10.1113/JP271684, PMID: 27027507 PMC5199745

[ref43] Convertino V. A. Lye K. R. Koons N. J. Joyner M. J. (2019). Physiological comparison of hemorrhagic shock and VO(2)max: a conceptual framework for defining the limitation of oxygen delivery. Exp. Biol. Med. 244, 690–701. doi: 10.1177/1535370219846425, PMID: 31042073 PMC6552402

[ref44] Convertino V. A. Rickards C. A. Lurie K. G. Ryan K. L. (2009). Hyperventilation in response to progressive reduction in central blood volume to near syncope. Aviat. Space Environ. Med. 80, 1012–1017. doi: 10.3357/ASEM.2598.2009, PMID: 20027847

[ref45] Coote J. H. (1995). Cardiovascular function of the paraventricular nucleus of the hypothalamus. Biol. Signals 4, 142–149. doi: 10.1159/000109434, PMID: 8750940

[ref46] Coote J. H. Spyer K. M. (2018). Central control of autonomic function. Brain Neurosci. Adv. 2:2398212818812012. doi: 10.1177/2398212818812012, PMID: 32166159 PMC7058216

[ref47] Corbani M. Marir R. Trueba M. Chafai M. Vincent A. Borie A. M.. (2018). Neuroanatomical distribution and function of the vasopressin V(1B) receptor in the rat brain deciphered using specific fluorescent ligands. Gen. Comp. Endocrinol. 258, 15–32. doi: 10.1016/j.ygcen.2017.10.011, PMID: 29155265

[ref48] Cowie M. R. Linz D. Redline S. Somers V. K. Simonds A. K. (2021). Sleep disordered breathing and cardiovascular disease: JACC state-of-the-art review. J. Am. Coll. Cardiol. 78, 608–624. doi: 10.1016/j.jacc.2021.05.048, PMID: 34353537

[ref49] Cregg J. M. Chu K. A. Dick T. E. Landmesser L. T. Silver J. (2017). Phasic inhibition as a mechanism for generation of rapid respiratory rhythms. Proc. Natl. Acad. Sci. U. S. A. 114, 12815–12820. doi: 10.1073/pnas.1711536114, PMID: 29133427 PMC5715763

[ref50] Currigan D. A. Hughes R. J. A. Wright C. E. Angus J. A. Soeding P. F. (2014). Vasoconstrictor responses to vasopressor agents in human pulmonary and radial arteries: an in vitro study. Anesthesiology 121, 930–936. doi: 10.1097/ALN.0000000000000430, PMID: 25198173

[ref52] da Silva E. F. Bassi M. Menani J. V. Colombari D. S. A. Zoccal D. B. Pedrino G. R.. (2019). Carotid bodies contribute to sympathoexcitation induced by acute salt overload. Exp. Physiol. 104, 15–27. doi: 10.1113/EP087110, PMID: 30370945 PMC6317332

[ref53] Doerschug K. C. Delsing A. S. Schmidt G. A. Ashare A. (2010). Renin-angiotensin system activation correlates with microvascular dysfunction in a prospective cohort study of clinical sepsis. Crit. Care 14:R24. doi: 10.1186/cc8887, PMID: 20175923 PMC2875539

[ref51] D’Silva J. L. Gill D. Mendel D. (1966). The effects of acute haemorrhage on respiration in the cat. J. Physiol. 187, 369–377. doi: 10.1113/jphysiol.1966.sp008096, PMID: 5972178 PMC1395939

[ref54] Dumais K. M. Veenema A. H. (2016). Vasopressin and oxytocin receptor systems in the brain: sex differences and sex-specific regulation of social behavior. Front. Neuroendocrinol. 40, 1–23. doi: 10.1016/j.yfrne.2015.04.003, PMID: 25951955 PMC4633405

[ref55] Düngen H.-D. Tscholl V. Obradovic D. Radenovic S. Matic D. Musial Bright L.. (2018). Prognostic performance of serial in-hospital measurements of copeptin and multiple novel biomarkers among patients with worsening heart failure: results from the MOLITOR study. ESC Hear. Fail. 5, 288–296. doi: 10.1002/ehf2.12231, PMID: 29476612 PMC5880673

[ref56] Enomoto M. Pan J. Shifrin Y. Belik J. (2014). Age dependency of vasopressin pulmonary vasodilatory effect in rats. Pediatr. Res. 75, 315–321. doi: 10.1038/pr.2013.221, PMID: 24257319 PMC3986081

[ref57] Evers K. S. Hügli M. Fouzas S. Kasser S. Pohl C. Stoecklin B.. (2020). Serum neurofilament levels in children with febrile seizures and in controls. Front. Neurosci. 14:579958. doi: 10.3389/fnins.2020.579958, PMID: 33132834 PMC7550525

[ref58] Ferguson A. V. Beckmann L. M. Fisher J. T. (1989). Effects of subfornical organ stimulation on respiration in the anesthetized rat. Can. J. Physiol. Pharmacol. 67, 1097–1101. doi: 10.1139/y89-173, PMID: 2598133

[ref59] Ferguson A. V. Latchford K. J. Samson W. K. (2008). The paraventricular nucleus of the hypothalamus—a potential target for integrative treatment of autonomic dysfunction. Expert Opin. Ther. Targets 12, 717–727. doi: 10.1517/14728222.12.6.717, PMID: 18479218 PMC2682920

[ref60] Finley J. C. Katz D. M. (1992). The central organization of carotid body afferent projections to the brainstem of the rat. Brain Res. 572, 108–116. doi: 10.1016/0006-8993(92)90458-L, PMID: 1611506

[ref61] Flôr A. F. L. de Brito Alves J. L. França-Silva M. S. Balarini C. M. Elias L. L. K. Ruginsk S. G.. (2018). Glial cells are involved in ANG-II-induced vasopressin release and sodium intake in awake rats. Front. Physiol. 9:430. doi: 10.3389/fphys.2018.00430, PMID: 29765330 PMC5938358

[ref62] Fortuna M. G. Kügler S. Hülsmann S. (2019). Probing the function of glycinergic neurons in the mouse respiratory network using optogenetics. Respir. Physiol. Neurobiol. 265, 141–152. doi: 10.1016/j.resp.2018.10.008, PMID: 30395936

[ref63] Frazier C. J. Harden S. W. Alleyne A. R. Mohammed M. Sheng W. Smith J. A.. (2021). An angiotensin-responsive connection from the lamina terminalis to the paraventricular nucleus of the hypothalamus evokes vasopressin secretion to increase blood pressure in mice. J. Neurosci. 41, 1429–1442. doi: 10.1523/JNEUROSCI.1600-20.2020, PMID: 33328294 PMC7896018

[ref64] Frier B. M. (2014). Hypoglycaemia in diabetes mellitus: epidemiology and clinical implications. Nat. Rev. Endocrinol. 10, 711–722. doi: 10.1038/nrendo.2014.170, PMID: 25287289

[ref65] Fyhrquist F. Eriksson L. Wallenius M. (1979). Plasma vasopressin in conscious goats after cerebroventricular infusions of angiotensins, sodium chloride, and fructose. Endocrinology 104, 1091–1095. doi: 10.1210/endo-104-4-1091, PMID: 436751

[ref66] Fyhrquist F. Tikkanen I. Linkola J. (1981). Plasma vasopressin concentration and renin in the rat: effect of hydration and hemorrhage. Acta Physiol. Scand. 113, 507–510. doi: 10.1111/j.1748-1716.1981.tb06929.x, PMID: 6753486

[ref67] Gallego R. Belmonte C. (1979). The effects of blood osmolality changes on cat carotid body chemoreceptors in vivo. Pflugers Arch. 380, 53–58. doi: 10.1007/BF00582612, PMID: 572038

[ref68] Gonzalez-Luque A. L’age M. Dhariwal A. P. Yates F. E. (1970). Stimulation of corticotropin release by corticotropin-releasing factor (CRF) or by vasopressin following intrapituitary infusions in unanesthetized dogs: inhibition of the responses by dexamethasone. Endocrinology 86, 1134–1142. doi: 10.1210/endo-86-5-1134, PMID: 4314067

[ref69] Gregoriano C. Molitor A. Haag E. Kutz A. Koch D. Haubitz S.. (2021). Activation of vasopressin system during COVID-19 is associated with adverse clinical outcomes: an observational study. J. Endocr. Soc. 5:bvab045. doi: 10.1210/jendso/bvab045, PMID: 34056499 PMC7989362

[ref70] Hanley D. F. Wilson D. A. Feldman M. A. Traystman R. J. (1988). Peripheral chemoreceptor control of neurohypophysial blood flow. Am. J. Physiol. 254, H742–H750. doi: 10.1152/ajpheart.1988.254.4.H742, PMID: 3128121

[ref71] Hasser E. M. Bishop V. S. (1990). Reflex effect of vasopressin after blockade of V1 receptors in the area postrema. Circ. Res. 67, 265–271. doi: 10.1161/01.RES.67.2.265, PMID: 2376071

[ref72] Heida J. E. Gansevoort R. T. Meijer E. (2021). Acid-base homeostasis during vasopressin V2 receptor antagonist treatment in autosomal dominant polycystic kidney disease patients. Kidney Int. Rep. 6, 839–841. doi: 10.1016/j.ekir.2020.12.021, PMID: 33732999 PMC7938056

[ref73] Hellenkamp K. Pruszczyk P. Jiménez D. Wyzgał A. Barrios D. Ciurzyński M.. (2018). Prognostic impact of copeptin in pulmonary embolism: a multicentre validation study. Eur. Respir. J. 51:1702037. doi: 10.1183/13993003.02037-2017, PMID: 29599188

[ref74] Henry J. L. Sessle B. J. (1989). Vasopressin and oxytocin express excitatory effects on respiratory and respiration-related neurones in the nuclei of the tractus solitarius in the cat. Brain Res. 491, 150–155. doi: 10.1016/0006-8993(89)90097-8, PMID: 2765879

[ref75] Hew-Butler T. Hoffman M. D. Stuempfle K. J. Rogers I. R. Morgenthaler N. G. Verbalis J. G. (2011). Changes in copeptin and bioactive vasopressin in runners with and without hyponatremia. Clin. J. Sport Med. 21, 211–217. doi: 10.1097/JSM.0b013e31821a62c2, PMID: 21519298 PMC3690462

[ref76] Hew-Butler T. Loi V. Pani A. Rosner M. H. (2017). Exercise-associated hyponatremia: 2017 update. Front. Med. 4:21. doi: 10.3389/fmed.2017.00021, PMID: 28316971 PMC5334560

[ref77] Hindmarch C. C. T. Fry M. Smith P. M. Yao S. T. Hazell G. G. J. Lolait S. J.. (2011). The transcriptome of the medullary area postrema: the thirsty rat, the hungry rat and the hypertensive rat. Exp. Physiol. 96, 495–504. doi: 10.1113/expphysiol.2010.056515, PMID: 21317217

[ref78] Hirasawa A. Hashimoto K. Tsujimoto G. (1994). Distribution and developmental change of vasopressin V1A and V2 receptor mRNA in rats. Eur. J. Pharmacol. 267, 71–75. doi: 10.1016/0922-4106(94)90226-7, PMID: 8206132

[ref79] Horstman L. L. Valle-Riestra B. J. Jy W. Wang F. Mao W. Ahn Y. S. (1995). Desmopressin (DDAVP) acts on platelets to generate platelet microparticles and enhanced procoagulant activity. Thromb. Res. 79, 163–174. doi: 10.1016/0049-3848(95)00102-W, PMID: 7676403

[ref80] Hume C. Allchorne A. Grinevich V. Leng G. Ludwig M. (2019). Effects of optogenetic stimulation of vasopressinergic retinal afferents on suprachiasmatic neurones. J. Neuroendocrinol. 31:e12806. doi: 10.1111/jne.12806, PMID: 31677199

[ref81] Hupf H. Grimm D. Riegger G. A. Schunkert H. (1999). Evidence for a vasopressin system in the rat heart. Circ. Res. 84, 365–370. doi: 10.1161/01.RES.84.3.365, PMID: 10024312

[ref82] Ikeda K. Igarashi H. Yawo H. Kobayashi K. Arata S. Kawakami K.. (2019). Optogenetic analysis of respiratory neuronal networks in the ventral medulla of neonatal rats producing channelrhodopsin in Phox2b-positive cells. Pflugers Arch. 471, 1419–1439. doi: 10.1007/s00424-019-02317-9, PMID: 31631251

[ref83] Imms F. J. Mehta D. (1989). Respiratory responses to sustained isometric muscle contractions in man: the effect of muscle mass. J. Physiol. 419, 1–14. doi: 10.1113/jphysiol.1989.sp017857, PMID: 2621624 PMC1189992

[ref84] Inder W. J. Hellemans J. Swanney M. P. Prickett T. C. Donald R. A. (1998). Prolonged exercise increases peripheral plasma ACTH, CRH, and AVP in male athletes. J. Appl. Physiol. 85, 835–841. doi: 10.1152/jappl.1998.85.3.835, PMID: 9729555

[ref85] Iovino M. Guastamacchia E. Giagulli V. A. Fiore G. Licchelli B. Iovino E.. (2013). Role of central and peripheral chemoreceptors in vasopressin secretion control. Endocr Metab Immune Disord Drug Targets 13, 250–255. doi: 10.2174/18715303113136660042, PMID: 24032393

[ref86] Ishii M. Hashimoto H. Ohkubo J.-I. Ohbuchi T. Saito T. Maruyama T.. (2016). Transgenic approach to express the channelrhodopsin 2 gene in arginine vasopressin neurons of rats. Neurosci. Lett. 630, 194–198. doi: 10.1016/j.neulet.2016.08.001, PMID: 27493075

[ref87] Jackson K. Silva H. M. V. Zhang W. Michelini L. C. Stern J. E. (2005). Exercise training differentially affects intrinsic excitability of autonomic and neuroendocrine neurons in the hypothalamic paraventricular nucleus. J. Neurophysiol. 94, 3211–3220. doi: 10.1152/jn.00277.2005, PMID: 16049146

[ref88] Japundzic-Zigon N. (2001). Effects of nonpeptide V1a and V2 antagonists on blood pressure fast oscillations in conscious rats. Clin. Exp. Hypertens. 23, 277–292. doi: 10.1081/CEH-100102667, PMID: 11349820

[ref89] Japundžić-Žigon N. Lozić M. Šarenac O. Murphy D. (2020). Vasopressin & oxytocin in control of the cardiovascular system: an updated review. Curr. Neuropharmacol. 18, 14–33. doi: 10.2174/1570159X17666190717150501, PMID: 31544693 PMC7327933

[ref90] Jennings D. B. (1994). The physicochemistry of [H+] and respiratory control: roles of PCO2, strong ions, and their hormonal regulators. Can. J. Physiol. Pharmacol. 72, 1499–1512. doi: 10.1139/y94-216, PMID: 7736341

[ref91] Jeon Y. Ryu J. H. Lim Y. J. Kim C. S. Bahk J.-H. Yoon S. Z.. (2006). Comparative hemodynamic effects of vasopressin and norepinephrine after milrinone-induced hypotension in off-pump coronary artery bypass surgical patients. Eur. J. Cardiothorac. Surg. 29, 952–956. doi: 10.1016/j.ejcts.2006.02.032, PMID: 16675238

[ref92] Johnson B. D. Saupe K. W. Dempsey J. A. (1992). Mechanical constraints on exercise hyperpnea in endurance athletes. J. Appl. Physiol. 73, 874–886. doi: 10.1152/jappl.1992.73.3.874, PMID: 1400051

[ref93] Johnson B. D. van Helmond N. Curry T. B. van Buskirk C. M. Convertino V. A. Joyner M. J. (2014). Reductions in central venous pressure by lower body negative pressure or blood loss elicit similar hemodynamic responses. J. Appl. Physiol. 117, 131–141. doi: 10.1152/japplphysiol.00070.2014, PMID: 24876357 PMC4459917

[ref94] Jurzak M. Schmid H. A. (1998). Vasopressin and sensory circumventricular organs. Prog. Brain Res. 119, 221–245. doi: 10.1016/s0079-6123(08)61572-1, PMID: 10074791

[ref95] Kakall Z. M. Cohen E. M. Farnham M. M. J. Kim S. J. Nedoboy P. E. Pilowsky P. M. (2019). Integration of hindbrain and carotid body mechanisms that control the autonomic response to cardiorespiratory and glucoprivic insults. Respir. Physiol. Neurobiol. 265, 83–91. doi: 10.1016/j.resp.2018.08.008, PMID: 30172780

[ref96] Kannan H. Hayashida Y. Yamashita H. (1989). Increase in sympathetic outflow by paraventricular nucleus stimulation in awake rats. Am. J. Physiol. 256, R1325–R1330. doi: 10.1152/ajpregu.1989.256.6.R1325, PMID: 2567578

[ref97] Kasserra C. E. Jones D. R. (1993). Hyperosmolality alters the ventilatory response to acute hypercapnia and hypoxia. Respir. Physiol. 94, 189–203. doi: 10.1016/0034-5687(93)90047-E, PMID: 8272590

[ref98] Kasserra C. E. Jones D. R. Hughes M. R. (1991). Acid-base disturbance and ventilatory response to changes in plasma osmolality in Pekin ducks. Respir. Physiol. 85, 383–393. doi: 10.1016/0034-5687(91)90076-U, PMID: 1961999

[ref99] Kc P. Balan K. V. Tjoe S. S. Martin R. J. Lamanna J. C. Haxhiu M. A.. (2010). Increased vasopressin transmission from the paraventricular nucleus to the rostral medulla augments cardiorespiratory outflow in chronic intermittent hypoxia-conditioned rats. J. Physiol. 588, 725–740. doi: 10.1113/jphysiol.2009.184580, PMID: 20051497 PMC2828143

[ref100] Kc P. Dick T. E. (2010). Modulation of cardiorespiratory function mediated by the paraventricular nucleus. Respir. Physiol. Neurobiol. 174, 55–64. doi: 10.1016/j.resp.2010.08.001, PMID: 20708107 PMC2967641

[ref101] Kc P. Haxhiu M. A. Tolentino-Silva F. P. Wu M. Trouth C. O. Mack S. O. (2002a). Paraventricular vasopressin-containing neurons project to brain stem and spinal cord respiratory-related sites. Respir. Physiol. Neurobiol. 133, 75–88. doi: 10.1016/s1569-9048(02)00131-3, PMID: 12385733

[ref102] Kc P. Haxhiu M. A. Trouth C. O. Balan K. V. Anderson W. A. Mack S. O. (2002b). CO(2)-induced c-Fos expression in hypothalamic vasopressin containing neurons. Respir. Physiol. 129, 289–296. doi: 10.1016/s0034-5687(01)00321-8, PMID: 11788132

[ref103] Kc P. Karibi-Ikiriko A. Rust C. F. Jayam-Trouth A. Haxhiu M. A. (2006). Phenotypic traits of the hypothalamic PVN cells innervating airway-related vagal preganglionic neurons. Respir. Physiol. Neurobiol. 154, 319–330. doi: 10.1016/j.resp.2006.01.006, PMID: 16515895 PMC1828905

[ref104] Knox A. J. Britton J. R. Tattersfield A. E. (1989). Effect of vasopressin on bronchial reactivity to histamine. Clin. Sci. 77, 467–471. doi: 10.1042/cs0770467, PMID: 2582720

[ref105] Koizumi H. Mosher B. Tariq M. F. Zhang R. Koshiya N. Smith J. C. (2016). Voltage-dependent rhythmogenic property of respiratory pre-bötzinger complex glutamatergic, Dbx1-derived, and somatostatin-expressing neuron populations revealed by graded optogenetic inhibition. eNeuro 3:ENEURO.0081–16.2016. doi: 10.1523/ENEURO.0081-16.2016, PMID: 27275007 PMC4891766

[ref106] Kolditz M. Halank M. Schulte-Hubbert B. Bergmann S. Albrecht S. Hoffken G. (2012). Copeptin predicts clinical deterioration and persistent instability in community-acquired pneumonia. Respir. Med. 106, 1320–1328. doi: 10.1016/j.rmed.2012.06.008, PMID: 22732597

[ref107] Koller E. A. Bührer A. Felder L. Schopen M. Vallotton M. B. (1991). Altitude diuresis: endocrine and renal responses to acute hypoxia of acclimatized and non-acclimatized subjects. Eur. J. Appl. Physiol. Occup. Physiol. 62, 228–234. doi: 10.1007/BF00643747, PMID: 2044531

[ref108] Koshimizu T. Nakamura K. Egashira N. Hiroyama M. Nonoguchi H. Tanoue A. (2012). Vasopressin V1a and V1b receptors: from molecules to physiological systems. Physiol. Rev. 92, 1813–1864. doi: 10.1152/physrev.00035.2011, PMID: 23073632

[ref109] Koyama Y. Coker R. H. Stone E. E. Lacy D. B. Jabbour K. Williams P. E.. (2000). Evidence that carotid bodies play an important role in glucoregulation in vivo. Diabetes 49, 1434–1442. doi: 10.2337/diabetes.49.9.1434, PMID: 10969826

[ref110] Kruger S. Ewig S. Giersdorf S. Hartmann O. Suttorp N. Welte T. (2010). Cardiovascular and inflammatory biomarkers to predict short- and long-term survival in community-acquired pneumonia: results from the German competence network, CAPNETZ. Am. J. Respir. Crit. Care Med. 182, 1426–1434. doi: 10.1164/rccm.201003-0415OC, PMID: 20639437

[ref111] Kuluöztürk M. İn E. Telo S. Karabulut E. Geçkil A. A. (2021). Efficacy of copeptin in distinguishing COVID-19 pneumonia from community-acquired pneumonia. J. Med. Virol. 93, 3113–3121. doi: 10.1002/jmv.26870, PMID: 33570194 PMC8013559

[ref112] Kumar P. Prabhakar N. R. (2012). Peripheral chemoreceptors: function and plasticity of the carotid body. Compr. Physiol. 2, 141–219. doi: 10.1002/cphy.c100069, PMID: 23728973 PMC3919066

[ref113] Landgraf R. Neumann I. D. (2004). Vasopressin and oxytocin release within the brain: a dynamic concept of multiple and variable modes of neuropeptide communication. Front. Neuroendocrinol. 25, 150–176. doi: 10.1016/j.yfrne.2004.05.001, PMID: 15589267

[ref114] Lang F. Busch G. L. Zempel G. Ditlevsen J. Hoch M. Emerich U.. (1995). Ca2+ entry and vasoconstriction during osmotic swelling of vascular smooth muscle cells. Pflugers Arch. 431, 253–258. doi: 10.1007/BF00410198, PMID: 9026786

[ref115] Launay J. M. Vittet D. Vidaud M. Rondot A. Mathieu M. N. Lalau-Keraly C.. (1987). V1a-vasopressin specific receptors on human platelets: potentiation by ADP and epinephrine and evidence for homologous down-regulation. Thromb. Res. 45, 323–331. doi: 10.1016/0049-3848(87)90221-0, PMID: 2953085

[ref116] Lee T. F. Mora F. Myers R. D. (1985). Effect of intracerebroventricular vasopressin on body temperature and endotoxin fever of macaque monkey. Am. J. Physiol. 248, R674–R678. doi: 10.1152/ajpregu.1985.248.6.R674, PMID: 4003578

[ref117] Lee A. K. Tse F. W. Tse A. (2015). Arginine vasopressin potentiates the stimulatory action of CRH on pituitary corticotropes via a protein kinase C-dependent reduction of the background TREK-1 current. Endocrinology 156, 3661–3672. doi: 10.1210/en.2015-1293, PMID: 26248219

[ref118] Levy M. N. (1966). Effect of carotid chemoreceptor stimulation on plasma antidiuretic hormone titer. Am. J. Physiol. 210, 157–161. doi: 10.1152/ajplegacy.1966.210.1.157

[ref119] Licis A. Davis G. Eisenstein S. A. Lugar H. M. Hershey T. (2019). Sleep disturbances in Wolfram syndrome. Orphanet J. Rare Dis. 14:188. doi: 10.1186/s13023-019-1160-z, PMID: 31375124 PMC6679489

[ref120] Lin Q. Fu F. Chen H. Zhu B. (2012). Copeptin in the assessment of acute lung injury and cardiogenic pulmonary edema. Respir. Med. 106, 1268–1277. doi: 10.1016/j.rmed.2012.05.010, PMID: 22728017

[ref121] Lipski J. McAllen R. M. Spyer K. M. (1977). The carotid chemoreceptor input to the respiratory neurones of the nucleus of tractus solitarus. J. Physiol. 269, 797–810. doi: 10.1113/jphysiol.1977.sp011930, PMID: 894614 PMC1283741

[ref122] Lipski J. McAllen R. M. Trzebski A. (1976). Carotid baroreceptor and chemoreceptor inputs onto single medullary neurones. Brain Res. 107, 132–136. doi: 10.1016/0006-8993(76)90101-3, PMID: 1268716

[ref123] Louwerse A. M. Marshall J. M. (1993). The role of vasopressin in the regional vascular responses evoked in the spontaneously breathing rat by systemic hypoxia. J. Physiol. 470, 463–472. doi: 10.1113/jphysiol.1993.sp019869, PMID: 8308738 PMC1143928

[ref124] Lüning H. Mangelus C. Carlström E. Nilson F. Börjesson M. (2019). Incidence and characteristics of severe exercise-associated collapse at the world’s largest half-marathon. PLoS One 14:e0217465. doi: 10.1371/journal.pone.0217465, PMID: 31173596 PMC6555510

[ref125] Maisel A. Xue Y. Shah K. Mueller C. Nowak R. Peacock W. F.. (2011). Increased 90-day mortality in patients with acute heart failure with elevated copeptin: secondary results from the biomarkers in acute heart failure (BACH) study. Circ. Heart Fail. 4, 613–620. doi: 10.1161/CIRCHEARTFAILURE.110.960096, PMID: 21765124

[ref126] Malheiros-Lima M. R. Totola L. T. Lana M. V. G. Strauss B. E. Takakura A. C. Moreira T. S. (2018). Breathing responses produced by optogenetic stimulation of adrenergic C1 neurons are dependent on the connection with preBötzinger complex in rats. Pflugers Arch. 470, 1659–1672. doi: 10.1007/s00424-018-2186-0, PMID: 30054719

[ref127] Marshall J. M. (1994). Peripheral chemoreceptors and cardiovascular regulation. Physiol. Rev. 74, 543–594. doi: 10.1152/physrev.1994.74.3.543, PMID: 8036247

[ref128] Matsuoka T. Saiki C. Mortola J. P. (1994). Metabolic and ventilatory responses to anemic hypoxia in conscious rats. J. Appl. Physiol. 77, 1067–1072. doi: 10.1152/jappl.1994.77.3.1067, PMID: 7836105

[ref129] McKinley M. J. Gerstberger R. Mathai M. L. Oldfield B. J. Schmid H. (1999). The lamina terminalis and its role in fluid and electrolyte homeostasis. J. Clin. Neurosci. 6, 289–301. doi: 10.1054/jocn.1998.0056, PMID: 10835180

[ref130] McMullan S. Dick T. E. Farnham M. M. J. Pilowsky P. M. (2009). Effects of baroreceptor activation on respiratory variability in rat. Respir. Physiol. Neurobiol. 166, 80–86. doi: 10.1016/j.resp.2009.02.006, PMID: 19429523 PMC2680772

[ref131] McMullan S. Pilowsky P. M. (2010). The effects of baroreceptor stimulation on central respiratory drive: a review. Respir. Physiol. Neurobiol. 174, 37–42. doi: 10.1016/j.resp.2010.07.009, PMID: 20674807

[ref132] Melo M. R. Gasparini S. Silva E. F. Karlen-Amarante M. Speretta G. F. Lauar M. R.. (2020). Renovascular hypertension elevates pulmonary ventilation in rats by carotid body-dependent mechanisms. Am. J. Phys. Regul. Integr. Comp. Phys. 318, R730–R742. doi: 10.1152/ajpregu.00134.2019, PMID: 32022595

[ref133] Milutinović S. Murphy D. Japundzić-Zigon N. (2006). The role of central vasopressin receptors in the modulation of autonomic cardiovascular controls: a spectral analysis study. Am. J. Phys. Regul. Integr. Comp. Phys. 291, R1579–R1591. doi: 10.1152/ajpregu.00764.2005, PMID: 17085750

[ref134] Moen V. Brudin L. Rundgren M. Irestedt L. (2014). Osmolality and respiratory regulation in humans: respiratory compensation for hyperchloremic metabolic acidosis is absent after infusion of hypertonic saline in healthy volunteers. Anesth. Analg. 119, 956–964. doi: 10.1213/ANE.0000000000000404, PMID: 25158789

[ref135] Mohan S. Moffett R. C. Thomas K. G. Irwin N. Flatt P. R. (2019). Vasopressin receptors in islets enhance glucose tolerance, pancreatic beta-cell secretory function, proliferation and survival. Biochimie 158, 191–198. doi: 10.1016/j.biochi.2019.01.008, PMID: 30677431

[ref136] Molnár Z. Petheo G. L. Fülöp C. Spät A. (2003). Effects of osmotic changes on the chemoreceptor cell of rat carotid body. J. Physiol. 546, 471–481. doi: 10.1113/jphysiol.2002.024125, PMID: 12527733 PMC2342532

[ref137] Montero S. A. Yarkov A. Lemus M. de Alvarez-Buylla E. R. Alvarez-Buylla R. (2006). Carotid chemoreceptor reflex modulation by arginine-vasopressin microinjected into the nucleus tractus solitarius in rats. Arch. Med. Res. 37, 709–716. doi: 10.1016/j.arcmed.2006.03.001, PMID: 16824929

[ref138] Morgenthaler N. G. Struck J. Alonso C. Bergmann A. (2006). Assay for the measurement of copeptin, a stable peptide derived from the precursor of vasopressin. Clin. Chem. 52, 112–119. doi: 10.1373/clinchem.2005.060038, PMID: 16269513

[ref139] Muller B. Morgenthaler N. Stolz D. Schuetz P. Muller C. Bingisser R.. (2007). Circulating levels of copeptin, a novel biomarker, in lower respiratory tract infections. Eur. J. Clin. Investig. 37, 145–152. doi: 10.1111/j.1365-2362.2007.01762.x, PMID: 17217381

[ref140] Murphy D. Konopacka A. Hindmarch C. Paton J. F. R. Sweedler J. V. Gillette M. U.. (2012). The hypothalamic-neurohypophyseal system: from genome to physiology. J. Neuroendocrinol. 24, 539–553. doi: 10.1111/j.1365-2826.2011.02241.x, PMID: 22448850 PMC3315060

[ref141] Nakamura K. Velho G. Bouby N. (2017). Vasopressin and metabolic disorders: translation from experimental models to clinical use. J. Intern. Med. 282, 298–309. doi: 10.1111/joim.12649, PMID: 28688111

[ref142] Nickel C. H. Bingisser R. Morgenthaler N. G. (2012). The role of copeptin as a diagnostic and prognostic biomarker for risk stratification in the emergency department. BMC Med. 10:7. doi: 10.1186/1741-7015-10-7, PMID: 22264220 PMC3275505

[ref143] Nickel N. Lichtinghagen R. Golpon H. Olsson K. M. Brand K. Welte T.. (2013). Circulating levels of copeptin predict outcome in patients with pulmonary arterial hypertension. Respir. Res. 14:130. doi: 10.1186/1465-9921-14-130, PMID: 24251953 PMC4176098

[ref144] Norsk P. (1989). Influence of low- and high-pressure baroreflexes on vasopressin release in humans. Acta Endocrinol. 121, 3–27. doi: 10.1530/acta.0.1210003-a, PMID: 2686333

[ref145] Nunn N. Womack M. Dart C. Barrett-Jolley R. (2011). Function and pharmacology of spinally-projecting sympathetic pre-autonomic neurones in the paraventricular nucleus of the hypothalamus. Curr. Neuropharmacol. 9, 262–277. doi: 10.2174/157015911795596531, PMID: 22131936 PMC3131718

[ref146] Nussey S. S. Prysor-Jones R. A. Taylor A. Ang V. T. Jenkins J. S. (1987). Arginine vasopressin and oxytocin in the bovine adrenal gland. J. Endocrinol. 115, 141–149. doi: 10.1677/joe.0.1150141, PMID: 3668443

[ref147] O’Connor M. D. Jennings D. B. (2001). Respiratory and metabolic effects of decreased osmolality in conscious rats. Can. J. Physiol. Pharmacol. 79, 768–778. doi: 10.1139/y01-056, PMID: 11599777

[ref148] Ohtake P. J. Jennings D. B. (1992). Ventilation is stimulated by small reductions in arterial pressure in the awake dog. J. Appl. Physiol. 73, 1549–1557. doi: 10.1152/jappl.1992.73.4.1549, PMID: 1447103

[ref149] Ohtake P. J. Jennings D. B. (1993). Angiotensin II stimulates respiration in awake dogs and antagonizes baroreceptor inhibition. Respir. Physiol. 91, 335–351. doi: 10.1016/0034-5687(93)90110-V, PMID: 8469855

[ref150] Oka Y. Ye M. Zuker C. S. (2015). Thirst driving and suppressing signals encoded by distinct neural populations in the brain. Nature 520, 349–352. doi: 10.1038/nature14108, PMID: 25624099 PMC4401619

[ref151] Ostrowski N. L. Lolait S. J. Young W. S. (1994). Cellular localization of vasopressin V1a receptor messenger ribonucleic acid in adult male rat brain, pineal, and brain vasculature. Endocrinology 135, 1511–1528. doi: 10.1210/endo.135.4.7925112, PMID: 7925112

[ref152] Overgaard C. B. Walker J. K. Jennings D. B. (1996). Respiration during acute hypoxia: angiotensin- and vasopressin-receptor blocks. J. Appl. Physiol. 80, 810–817. doi: 10.1152/jappl.1996.80.3.810, PMID: 8964741

[ref153] Pelletier J.-S. Dicken B. Bigam D. Cheung P.-Y. (2014). Cardiac effects of vasopressin. J. Cardiovasc. Pharmacol. 64, 100–107. doi: 10.1097/FJC.0000000000000092, PMID: 24621650

[ref154] Pittman Q. J. Franklin L. G. (1985). Vasopressin antagonist in nucleus tractus solitarius/vagal area reduces pressor and tachycardia responses to paraventricular nucleus stimulation in rats. Neurosci. Lett. 56, 155–160. doi: 10.1016/0304-3940(85)90122-3, PMID: 3925390

[ref155] Plotsky P. M. Bruhn T. O. Vale W. (1985). Hypophysiotropic regulation of adrenocorticotropin secretion in response to insulin-induced hypoglycemia. Endocrinology 117, 323–329. doi: 10.1210/endo-117-1-323, PMID: 2988921

[ref156] Pool A.-H. Wang T. Stafford D. A. Chance R. K. Lee S. Ngai J.. (2020). The cellular basis of distinct thirst modalities. Nature 588, 112–117. doi: 10.1038/s41586-020-2821-8, PMID: 33057193 PMC7718410

[ref157] Popovic M. Timper K. Seelig E. Nordmann T. Erlanger T. E. Donath M. Y.. (2019). Exercise upregulates copeptin levels which is not regulated by interleukin-1. PLoS One 14:e0217800. doi: 10.1371/journal.pone.0217800, PMID: 31150497 PMC6544286

[ref158] Potter E. K. McCloskey D. I. (1979). Respiratory stimulation by angiotensin II. Respir. Physiol. 36, 367–373. doi: 10.1016/0034-5687(79)90048-3, PMID: 441587

[ref159] Przybylski J. (1981). Do arterial chemoreceptors play a role in the pathogenesis of hypertension? Med. Hypotheses 7, 127–131. doi: 10.1016/0306-9877(81)90109-2, PMID: 7219239

[ref160] Pyner S. (2009). Neurochemistry of the paraventricular nucleus of the hypothalamus: implications for cardiovascular regulation. J. Chem. Neuroanat. 38, 197–208. doi: 10.1016/j.jchemneu.2009.03.005, PMID: 19778682

[ref161] Quillen E. W. J. Cowley A. W. J. (1983). Influence of volume changes on osmolality-vasopressin relationships in conscious dogs. Am. J. Physiol. 244, H73–H79. doi: 10.1152/ajpheart.1983.244.1.H73, PMID: 6849407

[ref162] Racca F. Vianello A. Mongini T. Ruggeri P. Versaci A. Vita G. L.. (2020). Practical approach to respiratory emergencies in neurological diseases. Neurol. Sci. 41, 497–508. doi: 10.1007/s10072-019-04163-0, PMID: 31792719 PMC7224095

[ref163] Raff H. (2011). Endocrine Adaptation to Hypoxia. Compr. Physiol. 1259–1275. doi: 10.1002/cphy.cp040254

[ref164] Raggenbass M. Tribollet E. Dubois-Dauphin M. Dreifuss J. J. (1989). Vasopressin receptors of the vasopressor (V1) type in the nucleus of the solitary tract of the rat mediate direct neuronal excitation. J. Neurosci. 9, 3929–3936. doi: 10.1523/JNEUROSCI.09-11-03929.1989, PMID: 2531217 PMC6569922

[ref165] Reddy M. K. Patel K. P. Schultz H. D. (2005). Differential role of the paraventricular nucleus of the hypothalamus in modulating the sympathoexcitatory component of peripheral and central chemoreflexes. Am. J. Phys. Regul. Integr. Comp. Phys. 289, R789–R797. doi: 10.1152/ajpregu.00222.2005, PMID: 15919733

[ref166] Richter D. W. Seller H. (1975). Baroreceptor effects on medullary respiratory neurones of the cat. Brain Res. 86, 168–171. doi: 10.1016/0006-8993(75)90651-4, PMID: 163666

[ref167] Sanada K. Yoshimura M. Ikeda N. Baba K. Nishimura H. Nishimura K.. (2021). Chemogenetic activation of endogenous arginine vasopressin exerts anorexigenic effects via central nesfatin-1/NucB2 pathway. J. Physiol. Sci. 71:18. doi: 10.1186/s12576-021-00802-4 34134629 PMC10717637

[ref168] Schill F. Timpka S. Nilsson P. M. Melander O. Enhörning S. (2021). Copeptin as a predictive marker of incident heart failure. ESC Hear. Fail. 8, 3180–3188. doi: 10.1002/ehf2.13439, PMID: 34056865 PMC8318511

[ref169] Schrier R. W. (2008). Vasopressin and aquaporin 2 in clinical disorders of water homeostasis. Semin. Nephrol. 28, 289–296. doi: 10.1016/j.semnephrol.2008.03.009, PMID: 18519089 PMC2587374

[ref170] Schrier R. W. Berl T. Anderson R. J. (1979). Osmotic and nonosmotic control of vasopressin release. Am. J. Physiol. 236, F321–F332. doi: 10.1152/ajprenal.1979.236.4.F321, PMID: 373467

[ref171] Scott C. S. Sharp-Kehl J. Redekopp C. A. Ledsome J. R. (1994). Regulation of plasma vasopressin by plasma osmolality and carotid sinus pressure in anesthetized rabbits. Am. J. Physiol. 266, R796–R801. doi: 10.1152/ajpregu.1994.266.3.R796, PMID: 8160873

[ref172] Senay L. C. J. (1969). Increased blood osmolarity and its effect on respiration of dehydrating men. Pflugers Arch. 309, 165–175. doi: 10.1007/BF00586966, PMID: 5815326

[ref173] Skytioti M. Søvik S. Elstad M. (2018). Respiratory pump maintains cardiac stroke volume during hypovolemia in young, healthy volunteers. J. Appl. Physiol. 124, 1319–1325. doi: 10.1152/japplphysiol.01009.2017, PMID: 29494288

[ref174] Smith P. M. Lowes V. L. Ferguson A. V. (1994). Circulating vasopressin influences area postrema neurons. Neuroscience 59, 185–194. doi: 10.1016/0306-4522(94)90109-0, PMID: 8190267

[ref175] Smith A. S. Williams Avram S. K. Cymerblit-Sabba A. Song J. Young W. S. (2016). Targeted activation of the hippocampal CA2 area strongly enhances social memory. Mol. Psychiatry 21, 1137–1144. doi: 10.1038/mp.2015.189, PMID: 26728562 PMC4935650

[ref176] Solaiman A. Z. Feehan R. P. Chabitnoy A. M. Leuenberger U. A. Monahan K. D. (2014). Ventilatory responses to chemoreflex stimulation are not enhanced by angiotensin II in healthy humans. Auton. Neurosci. 183, 72–79. doi: 10.1016/j.autneu.2014.01.010, PMID: 24556416 PMC4058361

[ref177] Souza G. M. P. R. Stornetta R. L. Stornetta D. S. Abbott S. B. G. Guyenet P. G. (2020). Differential contribution of the retrotrapezoid nucleus and C1 neurons to active expiration and arousal in rats. J. Neurosci. 40, 8683–8697. doi: 10.1523/JNEUROSCI.1006-20.2020, PMID: 32973046 PMC7643293

[ref178] Srinivasan M. Bongianni F. Fontana G. A. Pantaleo T. (1993). Respiratory responses to electrical and chemical stimulation of the area postrema in the rabbit. J. Physiol. 463, 409–420. doi: 10.1113/jphysiol.1993.sp019601, PMID: 8246191 PMC1175350

[ref179] Sugawara Y. Mizuno Y. Oku S. Goto T. (2019). Effects of vasopressin during a pulmonary hypertensive crisis induced by acute hypoxia in a rat model of pulmonary hypertension. Br. J. Anaesth. 122, 437–447. doi: 10.1016/j.bja.2019.01.014, PMID: 30857600 PMC6435915

[ref180] Szczepańska-Sadowska E. (1996). Interaction of vasopressin and angiotensin II in central control of blood pressure and thirst. Regul. Pept. 66, 65–71. doi: 10.1016/0167-0115(96)00053-5, PMID: 8899896

[ref181] Szczepanska-Sadowska E. Czarzasta K. Cudnoch-Jedrzejewska A. (2018). Dysregulation of the renin-angiotensin system and the vasopressinergic system interactions in cardiovascular disorders. Curr. Hypertens. Rep. 20:19. doi: 10.1007/s11906-018-0823-9, PMID: 29556787 PMC5859051

[ref182] Szczepanska-Sadowska E. Zera T. Sosnowski P. Cudnoch-Jedrzejewska A. Puszko A. Misicka A. (2017). Vasopressin and related peptides; potential value in diagnosis, prognosis and treatment of clinical disorders. Curr. Drug Metab. 18, 306–345. doi: 10.2174/1389200218666170119145900, PMID: 28117000

[ref183] Szmydynger-Chodobska J. Chung I. Kozniewska E. Tran B. Harrington F. J. Duncan J. A.. (2004). Increased expression of vasopressin v1a receptors after traumatic brain injury. J. Neurotrauma 21, 1090–1102. doi: 10.1089/0897715041651033, PMID: 15319008

[ref184] Sztechman D. Czarzasta K. Cudnoch-Jedrzejewska A. Szczepanska-Sadowska E. Zera T. (2018). Aldosterone and mineralocorticoid receptors in regulation of the cardiovascular system and pathological remodelling of the heart and arteries. J. Physiol. Pharmacol. 69, 829–845. doi: 10.26402/jpp.2018.6.01, PMID: 30898981

[ref185] Tabarean I. V. (2021). Activation of preoptic arginine vasopressin neurons induces hyperthermia in male mice. Endocrinology 162:bqaa217. doi: 10.1210/endocr/bqaa217, PMID: 33249461 PMC7758908

[ref186] Tahara A. Tomura Y. Wada K. Kusayama T. Tsukada J. Ishii N.. (1998). Characterization of vasopressin receptor in rat lung. Neuropeptides 32, 281–286. doi: 10.1016/S0143-4179(98)90049-X, PMID: 10189064

[ref187] Takamata A. Nose H. Kinoshita T. Hirose M. Itoh T. Morimoto T. (2000). Effect of acute hypoxia on vasopressin release and intravascular fluid during dynamic exercise in humans. Am. J. Phys. Regul. Integr. Comp. Phys. 279, R161–R168. doi: 10.1152/ajpregu.2000.279.1.R161, PMID: 10896878

[ref188] Takeda M. Dubey R. Phillips J. K. Matsumoto S. Lipski J. (2002). Effects of vasopressin on isolated rat adrenal chromaffin cells. Regul. Pept. 106, 55–65. doi: 10.1016/S0167-0115(02)00036-8, PMID: 12047911

[ref189] Thompson C. J. Charlton J. Walford S. Baird J. Hearnshaw J. McCulloch A.. (1989). Vasopressin secretion in the DIDMOAD (Wolfram) syndrome. Q. J. Med. 71, 333–345. PMID: 2687931

[ref190] Thompson E. L. Ray C. J. Holmes A. P. Pye R. L. Wyatt C. N. Coney A. M.. (2016). Adrenaline release evokes hyperpnoea and an increase in ventilatory CO_2_ sensitivity during hypoglycaemia: a role for the carotid body. J. Physiol. 594, 4439–4452. doi: 10.1113/JP272191, PMID: 27027261 PMC4967760

[ref191] Thornton S. N. Leng G. Bicknell R. J. Chapman C. Purdew T. (1986). Vasopressin, but not oxytocin, is released in response to water deprivation in conscious goats. J. Endocrinol. 110, 335–340. doi: 10.1677/joe.0.1100335, PMID: 3746167

[ref192] Tribollet E. Raufaste D. Maffrand J. Serradeil-Le Gal C. (1999). Binding of the non-peptide vasopressin V1a receptor antagonist SR-49059 in the rat brain: an in vitro and in vivo autoradiographic study. Neuroendocrinology 69, 113–120. doi: 10.1159/000054409, PMID: 9986924

[ref193] Trzebski A. Chruścielewski L. Majcherczyk S. (1978). Effect of osmotic stimuli on the carotid baroreceptor and chemoreceptor discharges in cats. Acta Phys. Pol. A 29, 373–377. PMID: 742372

[ref194] Ueta Y. Dayanithi G. Fujihara H. (2011). Hypothalamic vasopressin response to stress and various physiological stimuli: visualization in transgenic animal models. Horm. Behav. 59, 221–226. doi: 10.1016/j.yhbeh.2010.12.007, PMID: 21185297

[ref195] Ufnal M. Skrzypecki J. (2014). Blood borne hormones in a cross-talk between peripheral and brain mechanisms regulating blood pressure, the role of circumventricular organs. Neuropeptides 48, 65–73. doi: 10.1016/j.npep.2014.01.003, PMID: 24485840

[ref196] Venus B. Mathru M. Smith R. A. Pham C. G. Shirakawa Y. Sugiura A. (1985). Renal function during application of positive end-expiratory pressure in swine: effects of hydration. Anesthesiology 62, 765–769. doi: 10.1097/00000542-198506000-00011, PMID: 3890619

[ref197] Verbalis J. G. (2007). How does the brain sense osmolality? J. Am. Soc. Nephrol. 18, 3056–3059. doi: 10.1681/ASN.2007070825, PMID: 18003769

[ref198] Verney E. B. (1947). The antidiuretic hormone and the factors which determine its release. Proc. R. Soc. Lond. Ser. B Biol. Sci. 135, 25–106. PMID: 18918876

[ref199] Wade C. E. Claybaugh J. R. (1980). Plasma renin activity, vasopressin concentration, and urinary excretory responses to exercise in men. J. Appl. Physiol. 49, 930–936. doi: 10.1152/jappl.1980.49.6.930, PMID: 7002889

[ref200] Wade J. B. Stetson D. L. Lewis S. A. (1981). ADH action: evidence for a membrane shuttle mechanism. Ann. N. Y. Acad. Sci. 372, 106–117. doi: 10.1111/j.1749-6632.1981.tb15464.x, PMID: 6951416

[ref201] Walker B. R. (1986). Role of vasopressin in the cardiovascular response to hypoxia in the conscious rat. Am. J. Physiol. 251, H1316–H1323. doi: 10.1152/ajpheart.1986.251.6.H1316, PMID: 3098115

[ref202] Walker B. R. Haynes J. J. Wang H. L. Voelkel N. F. (1989). Vasopressin-induced pulmonary vasodilation in rats. Am. J. Physiol. 257, H415–H422. doi: 10.1152/ajpheart.1989.257.2.H415, PMID: 2764128

[ref203] Walker J. K. Jennings D. B. (1994). Angiotensin mediates stimulation of ventilation after vasopressin V1 receptor blockade. J. Appl. Physiol. 76, 2517–2526. doi: 10.1152/jappl.1994.76.6.2517, PMID: 7928878

[ref204] Walker J. K. Jennings D. B. (1995). During acute hypercapnia vasopressin inhibits an angiotensin drive to ventilation in conscious dogs. J. Appl. Physiol. 79, 786–794. doi: 10.1152/jappl.1995.79.3.786, PMID: 8567518

[ref205] Walker J. K. Jennings D. B. (1996). Ventilatory effects of angiotensin and vasopressin in conscious rats. Can. J. Physiol. Pharmacol. 74, 1258–1264. doi: 10.1139/y96-140, PMID: 9028585

[ref206] Walker J. K. Jennings D. B. (1998). Respiratory effects of pressor and depressor agents in conscious rats. Can. J. Physiol. Pharmacol. 76, 707–714. doi: 10.1139/y98-081, PMID: 10030450

[ref207] Wallace A. W. Tunin C. M. Shoukas A. A. (1989). Effects of vasopressin on pulmonary and systemic vascular mechanics. Am. J. Physiol. 257, H1228–H1234. doi: 10.1152/ajpheart.1989.257.4.H1228, PMID: 2801982

[ref208] Wang B. C. Sundet W. D. Goetz K. L. (1984). Vasopressin in plasma and cerebrospinal fluid of dogs during hypoxia or acidosis. Am. J. Physiol. 247, E449–E455. doi: 10.1152/ajpendo.1984.247.4.E449, PMID: 6496666

[ref209] Ward D. S. Voter W. A. Karan S. (2007). The effects of hypo- and hyperglycaemia on the hypoxic ventilatory response in humans. J. Physiol. 582, 859–869. doi: 10.1113/jphysiol.2007.130112, PMID: 17478538 PMC2075331

[ref210] Wehberg K. E. Gala G. J. Brunner M. J. (1991). Comparison of carotid baroreflex control of plasma AVP concentration in conscious and anesthetized dogs. Am. J. Physiol. 261, R950–R956. doi: 10.1152/ajpregu.1991.261.4.R950, PMID: 1928441

[ref211] Wehrwein E. A. Limberg J. K. Taylor J. L. Dube S. Basu A. Basu R.. (2015). Effect of bilateral carotid body resection on the counterregulatory response to hypoglycaemia in humans. Exp. Physiol. 100, 69–78. doi: 10.1113/expphysiol.2014.083154, PMID: 25557731

[ref212] Wilson D. A. Hanley D. F. Feldman M. A. Traystman R. J. (1987). Influence of chemoreceptors on neurohypophyseal blood flow during hypoxic hypoxia. Circ. Res. 61, 194–101. PMID: 3664988

[ref213] Yan X. Chen X. Guo Y. He D. Chen Y. Xia C.. (2017). Arginine vasopressin alters both spontaneous and phase-locked synaptic inputs to airway vagal preganglionic neuron via activation of V(1a) receptor: insights into stress-related airway vagal excitation. Front. Cell. Neurosci. 11:12. doi: 10.3389/fncel.2017.00012, PMID: 28210214 PMC5288349

[ref214] Yang Z. Coote J. H. (1998). Influence of the hypothalamic paraventricular nucleus on cardiovascular neurones in the rostral ventrolateral medulla of the rat. J. Physiol. 513, 521–530. doi: 10.1111/j.1469-7793.1998.521bb.x, PMID: 9807000 PMC2231294

[ref215] Yang S.-J. Lee K.-Z. Wu C.-H. Lu K.-T. Hwang J.-C. (2006). Vasopressin produces inhibition on phrenic nerve activity and apnea through V(1A) receptors in the area postrema in rats. Chin. J. Phys. 49, 313–325. PMID: 17357538

[ref216] Yang Z. Wheatley M. Coote J. H. (2002). Neuropeptides, amines and amino acids as mediators of the sympathetic effects of paraventricular nucleus activation in the rat. Exp. Physiol. 87, 663–674. doi: 10.1113/eph8702439, PMID: 12530399

[ref217] Yang D.-B. Yu W.-H. Dong X.-Q. Du Q. Shen Y.-F. Zhang Z.-Y.. (2014). Plasma copeptin level predicts acute traumatic coagulopathy and progressive hemorrhagic injury after traumatic brain injury. Peptides 58, 26–29. doi: 10.1016/j.peptides.2014.05.015, PMID: 24905622

[ref218] Yeung M. L. Teng J. L. L. Jia L. Zhang C. Huang C. Cai J.-P.. (2021). Soluble ACE2-mediated cell entry of SARS-CoV-2 via interaction with proteins related to the renin-angiotensin system. Cell 184, 2212.e12–2228.e12. doi: 10.1016/j.cell.2021.02.053, PMID: 33713620 PMC7923941

[ref219] Yoshimura M. Nishimura K. Nishimura H. Sonoda S. Ueno H. Motojima Y.. (2017). Activation of endogenous arginine vasopressin neurons inhibit food intake: by using a novel transgenic rat line with DREADDs system. Sci. Rep. 7:15728. doi: 10.1038/s41598-017-16049-2, PMID: 29146932 PMC5691068

[ref220] Yoshimura M. Ueta Y. (2019). Advanced genetic and viral methods for labelling and manipulation of oxytocin and vasopressin neurones in rats. Cell Tissue Res. 375, 311–327. doi: 10.1007/s00441-018-2932-9, PMID: 30338378

[ref221] Zera T. Moraes D. J. A. da Silva M. P. Fisher J. P. Paton J. F. R. (2019). The logic of carotid body connectivity to the brain. Physiology 34, 264–282. doi: 10.1152/physiol.00057.2018, PMID: 31165684

[ref222] Żera T. Przybylski J. Grygorowicz T. Kasarełło K. Podobińska M. Mirowska-Guzel D.. (2018). Vasopressin V1a receptors are present in the carotid body and contribute to the control of breathing in male Sprague-Dawley rats. Peptides 102, 68–74. doi: 10.1016/j.peptides.2018.03.004, PMID: 29524562

[ref223] Zerbe R. L. Feuerstein G. (1985). Cardiovascular effects of centrally administered vasopressin in conscious and anesthetized rats. Neuropeptides 6, 471–483. doi: 10.1016/0143-4179(85)90146-5, PMID: 4058686

[ref224] Zhang Z.-Y. Zhang L.-X. Dong X.-Q. Yu W.-H. Du Q. Yang D.-B.. (2014). Comparison of the performances of copeptin and multiple biomarkers in long-term prognosis of severe traumatic brain injury. Peptides 60, 13–17. doi: 10.1016/j.peptides.2014.07.016, PMID: 25076464

[ref225] Zhao Y. Jiang Y. Zhou L. Wu X. (2014). The value of assessment tests in patients with acute exacerbation of chronic obstructive pulmonary disease. Am. J. Med. Sci. 347, 393–399. doi: 10.1097/MAJ.0b013e31829a63b1, PMID: 24270077

[ref226] Zheng F. Copotoiu R. Tacquard C. Demoulin B. Malinovsky J. M. Levy B.. (2017). Epinephrine but not vasopressin attenuates the airway response to anaphylactic shock in rats. Exp. Lung Res. 43, 158–166. doi: 10.1080/01902148.2017.1323981, PMID: 28541755

[ref227] Zhou T. Chien M.-S. Kaleem S. Matsunami H. (2016). Single cell transcriptome analysis of mouse carotid body glomus cells. J. Physiol. 594, 4225–4251. doi: 10.1113/JP271936, PMID: 26940531 PMC4967736

[ref228] Zimmerman C. A. Lin Y.-C. Leib D. E. Guo L. Huey E. L. Daly G. E.. (2016). Thirst neurons anticipate the homeostatic consequences of eating and drinking. Nature 537, 680–684. doi: 10.1038/nature18950, PMID: 27487211 PMC5161740

